# Transcriptome analysis identifies candidate genes in the biosynthetic pathway of sex pheromones from a zygaenid moth, *Achelura yunnanensis* (Lepidoptera: Zygaenidae)

**DOI:** 10.7717/peerj.12641

**Published:** 2021-12-14

**Authors:** Shu-Mei Nuo, An-Jin Yang, Gen-Ceng Li, Hai-Yan Xiao, Nai-Yong Liu

**Affiliations:** Key Laboratory of Forest Disaster Warning and Control of Yunnan Province, Southwest Forestry University, Kunming, Yunnan, China

**Keywords:** *Achelura yunnanensis*, Pheromone gland, Transcriptome, Pheromone biosynthesis gene, Pheromone degradation gene

## Abstract

In most moth species, sex pheromones responsible for mating and communication of both sexes are primarily produced by the pheromone glands (PGs) of female moths. Although the PG transcriptomes and pheromone production related genes from 24 moth species have been characterized, studies on the related information remain unknown in the Zygaenidae family. Here, we sequenced the PG transcriptome of a zygaenid moth, *Achelura yunnanensis*. Such the sequencing resulted in the yields of 47,632,610 clean reads that were assembled into 54,297 unigenes, coupled with RNA sequencing data from 12 other tissues. Based on the transcriptome, a total of 191 genes encoding pheromone biosynthesis and degradation enzymes were identified, 161 of which were predicted to have full-length sequences. A comparative analysis among 24 moth species of nine families indicated that the numbers of the genes were variable, ranging from 14 in two *Grapholita* species to 191 in *A. yunnanensis*. Phylogenetic analysis in parallel with the expression data highlighted some key genes, including three △9 and four △11 desaturases, four fatty acyl-CoA reductases (FARs) clustering in the pgFAR clade, and three significantly antennae-enriched aldehyde oxidases. An extensive tissue- and sex- expression profile revealed a broad distribution of the genes, in which 128 relatives were detected in the PGs and 127 in the antennae. This study reports, for the first time, the gene repertoires associated with the pheromone production in Zygaenidae, and provides a valuable resource for exploring putative roles of the PG-enriched genes in *A. yunnanensis*.

## Introduction

The chemical communication among moths is mediated primarily by female sex pheromones released by a differentiated tissue, pheromone gland (PG). The sex pheromones produced by the PGs are generally a blend of multiple components, with a unique ratio serving as species recognition (intraspecific communication) and reproductive isolation (interspecific communication) (Tillman et al., 1999; Raina et al., 2000; Jurenka, 2021). Thus, it is of particular significance to understand how the female moths biosynthesize sex pheromones in the PGs, and to identify the genes associated with the pheromone production and degradation. To date, the PG transcriptomes of at least 25 moths from 10 families have been sequenced and characterized, including a model lepidopteran moth *Bombyx mori* and *Achelura yunnanensis* presented in this study (see Table 1; Zhang et al., 2014a; Jurenka, 2021). However, in the Zygaenidae family the transcriptomic data and related genes in the PG tissues have not been reported, restricting our knowledge of sex pheromone evolution and genetic variation across zygaenid moths.

**Table 1:** Comparisons of candidate genes involved in the sex pheromone biosynthesis among moths. Asterisks indicate that the gene families were not reported from the transcriptomes. Aips, *Agrotis ipsilon* (Gu et al., 2013); Aper, *Antheraea pernyi* (Wang et al., 2020); Aseg, *Agrotis segetum* (Ding & Lofstedt, 2015); Ayun, *Achelura yunnanensis*; Cher, *Ctenopseutis herana* (Grapputo et al., 2018); Cobl, *Ctenopseutis obliquana* (Grapputo et al., 2018); Csup, *Chilo suppressalis* (Xia et al., 2015); Ecau, *Ephestia cautella* (Antony et al., 2015); Gdim, *Grapholita dimorpha* (Jung & Kim, 2014); Gmol, *Grapholita molesta* (Jung & Kim, 2014); Hass, *Helicoverpa assulta* (Li et al., 2015); Harm, *Helicoverpa armigera* (Li et al., 2015); Hvir, *Heliothis virescens* (Vogel et al., 2010); Hzea, *Helicoverpa zea* (Dou et al., 2019a); Mvit, *Maruca vitrata* (Cha et al., 2017); Nbil, *Noorda blitealis* (Zhang et al., 2021); Pexc, *Planotortrix excessana* (Grapputo et al., 2018); Pgos, *Pectinophora gossypiella* (Dou et al., 2019b); Poct, *Planotortrix octo* (Grapputo et al., 2018); Pxyl, *Plutella xylostella* (He et al., 2017); Sexi, *Spodoptera exigua* (Zhang et al., 2017); Sinf, *Sesamia inferens* (Zhang et al., 2014a; Zhang et al., 2014ab); Slit, *Spodoptera litura* (Zhang et al., 2015) and Sins, *Streltzoviella insularis* (Yang, Tao & Zong, 2020).

Species	ACC	FAS	FAD	FAR	AR	ALDH	AO/ADH	ATF	FATP	AOX	Total	Transcriptome
**Zygaenidae**													
*Ayun*	3	12	22	24	10	13	34	63	4	6	191	PG and 12 other tissues
**Noctuidae**													
*Aips*	2	1	5	13	11	*	5	5	*	*	42	PG
*Aseg*	1	6	10	10	*	*	*	34	*	*	61	PG and bodies
*Harm*	2	3	7	18	*	9	17	*	*	7	63	PG
*Hass*	2	3	8	13	*	6	18	*	*	4	54	PG
*Hvir*	1	3	9	5	2	8	5	3	*	6	42	PG
*Hzea*	1	3	9	20	*	4	*	*	*	*	37	PG and tarsi
*Sexi*	1	*	10	13	*	*	*	29	4	*	57	PG
*Slit*	1	*	12	13	*	*	*	23	4	*	53	PG
*Sinf*	7	*	6	3	6	*	6	5	5	3	41	PG and antennae
**Tortricidae**													
*Gdim*	4	2	4	1	2	*	*	1	*	*	14	PG
*Gmol*	4	2	4	1	2	*	*	1	*	*	14	PG
*Cher*	1	1	6	15	*	*	*	17	4	*	44	PG and antennae
*Cobl*	1	1	7	15	*	*	*	21	4	*	49	PG and antennae
*Pexc*	1	1	6	15	*	*	*	21	4	*	48	PG and antennae
*Poct*	1	1	6	15	*	*	*	21	4	*	48	PG and antennae
**Pyralidae**													
*Csup*	1	1	6	10	*	*	*	*	4	1	23	PG
*Ecau*	6	12	22	28	11	*	*	18	5	*	102	PG
**Crambidae**													
*Mvit*	*	29	20	4	*	*	5	6	*	*	64	PG
*Nbil*	2	16	12	13	3	10	24	28	*	6	114	PG and bodies
**Cossidae**													
*Sins*	2	5	17	13	5	*	5	2	*	*	49	PG
**Plutellidae**													
*Pxyl*	7	7	15	7	13	5	21	18	3	3	99	PG and five other tissues
**Saturniidae**													
*Aper*	1	1	6	11	7	*	24	22	4	1	77	PG
**Gelechiidae**													
*Pgos*	1	4	17	8	*	*	*	17	*	*	47	PG

In line with other most moths, female moths in Zygaenidae primarily synthesize the Type I sex pheromones (Löfstedt, Wahlberg & Millar, 2016; Yuvaraj et al., 2017). Therefore, they have similar fatty acid biosynthesis pathways with the majority of moth species, in which the palmitic (C16) and/or stearic (C18) acids are converted into specific chain-length (C10–C18) fatty acid derivatives with one or more double bonds (alcohols, aldehydes and acetate esters) *via* a series of enzymatic reactions, including fatty acid biosynthesis, desaturation, chain-shortening, reduction and acetylation (Roelofs & Rooney, 2003; Ando, Inomata & Yamamoto, 2004; Jurenka, 2004). As the first step of pheromone biosynthesis, two enzymes of acetyl-CoA carboxylases (ACCs) and fatty acid synthases (FASs) are involved in the synthesis of saturated fatty acids, the precursors of sex pheromones (Volpe & Vagelos, 1973; Pape, Lopez-Casillas & Kim, 1988; Foster & Anderson, 2012). Next, members of a key gene family encoding fatty acyl desaturases (FADs) determine the diversity of pheromone structures by introducing double bonds into specific positions of sex pheromones, largely contributing to the specificity of sex pheromone communication (Hao et al., 2002; Knipple et al., 2002; Fujii et al., 2015; Xia et al., 2019). In some case, the long-chain unsaturated fatty acids are shortened by *β*-oxidation enzymes to further increase the diversity of species-specific pheromones (Bjostad, Wolf & Roelofs, 1987). Lastly, in most moths a terminal functional group (–OH, –Ald or –OAc) is formed by fatty acyl-CoA reductases (FARs), alcohol oxidases/dehydrogenases (AOs/ADHs) or acetyltransferases (ATFs) (Teal & Tumlinson, 1986; Morse & Meighen, 1987; Moto et al., 2003; Lienard et al., 2010; Hagstrom et al., 2012; Jurenka, 2021).

Except for the biosynthesis of sex pheromones, the pheromone degradation also requires a variety of enzymes such as aldehyde oxidases (AOXs). In Lepidoptera, the AOX gene family comprises six conserved groups (AOX1–AOX6), in which a novel clade (AOX6) was more recently proposed (Xu & Liao, 2017; Zhang et al., 2021). Functional experiments have demonstrated that AOXs enriched in moth antennae could degrade aldehyde compounds into carboxylic acids, including aldehyde-containing sex pheromones, plant volatiles and insecticides (Choo et al., 2013; Wang et al., 2021). However, none of AOXs in the PGs have been functionally characterized to date.

The zygaenid moth, *A. yunnanensis* (Lepidoptera: Zygaenidae), is a destructive garden pest with the larvae feeding on the leaves of the Rosaceae plants, including *Photinia glomerata*, *Chaenomeles sinensis* and *Cerasus yunnanensis*. Recently, the sensilla on chemosensory tissues and the genes involved in chemosensation in this species have been characterized (Li et al., 2020; Li et al., 2021), greatly enhancing our understanding of intra- and interspecific interactions. Notably, although the PG transcriptomes of 24 moth species are available (see Table 1; Zhang et al., 2014a; Jurenka, 2021), in Zygaenidae the related transcriptomes and the genes have not yet been surveyed to date. Here, we sequenced and assembled the transcriptome of the PGs and 12 other tissues previously sequenced (Li et al., 2021). Based on the transcriptomic assembly in this species, we identified and characterized the genes responsible for the biosynthesis and degradation of sex pheromones, which were categorized into 10 enzyme gene families. This study will assist in, to some extent, the identification of sex pheromones in *A. yunnanensis*, and complements the information of the pheromone biosynthesis and degradation genes in zygaenid moths.

## Materials and Methods

### Insects and tissues

The larvae of *A. yunnanensis* were reared as previously described by Li et al. (2021). After emergence, female and male adults were distinguished according to the genitalia, and were maintained separately in independent cages with 10% honey solution.

**Figure 1:**
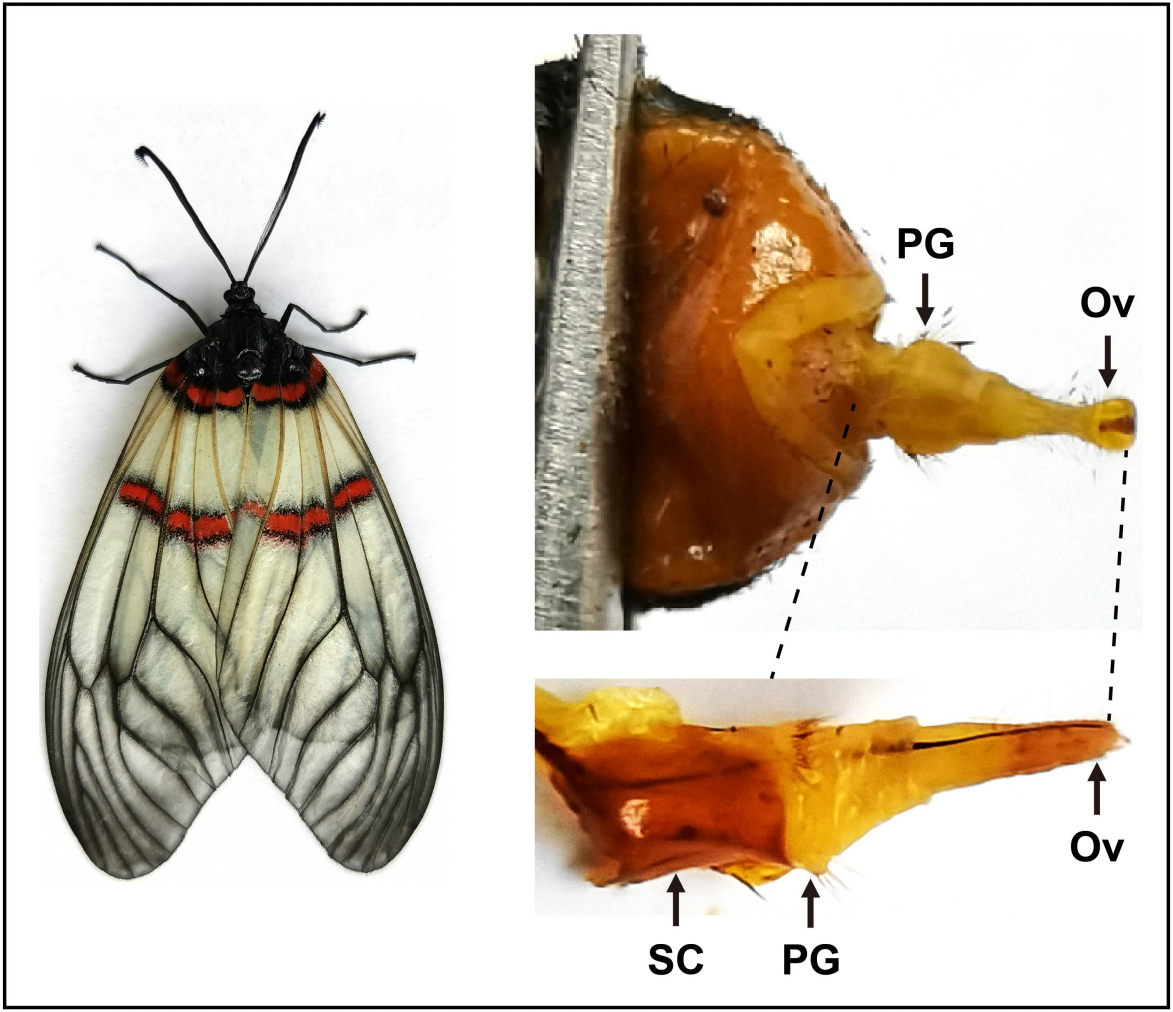
Pictures of the female moth and the pheromone gland in *A. yunnanensis*. In RNA-Seq and expression profiling analyses, the sclerotized cuticles (SC) were removed. PG, pheromone gland and Ov, sclerotized ovipositor valve.

To sequence the PG tissues and investigate expression profiles of genes, we dissected the PGs and other tissues from larvae and adults. For transcriptome sequencing, approximately 30 PGs were pooled from 3–day–old virgin female moths. As indicated in the previous studies (Vogel et al., 2010; Li et al., 2015; Xia et al., 2015), here the sclerotized cuticles of PGs in *A. yunnanensis* were first cleaned, and the rest tissues with ovipositors were used for the construction of the RNA–Seq library (Fig. 1). In the expression profiling analyses of genes, each tissue comprised three biological samples from 3–day–old adults and 4th instar larvae. Each biological sample was composed of 50 antennae, 30 heads without antennae, five thoraxes, five abdomens without PGs, 20 legs or 20 wings of both sexes, as well as 300 larval antennae or 300 larval maxillary palps, respectively.

### *De novo* transcriptome sequencing, assembly and analysis

First, we isolated total RNA of *A. yunnanensis* PGs using TRIzol reagent (Ambion, Life Technologies, Carlsbad, CA, USA), according to the manufacturer’s instructions. Genomic DNA (gDNA) of total RNA samples was digested with treatment of DNase I (Amplification Grade; Invitrogen Life Technologies, Carlsbad, CA, USA). Next, the purity, concentration and integrity of RNA were evaluated by a NanoDrop spectrophotometer (Thermo Fisher Scientific, Waltham, MA, USA), a Qubit 2.0 Fluorometer (Invitrogen Life Technologies, Waltham, MA, USA) and an Agilent 2100 Bioanalyzer (Agilent Technologies, Santa Clara, CA, USA), respectively. The RNA–Seq library was constructed using the NEBNext^^®^^ Ultra™ RNA Library Prep Kit for Illumina^^®^^ (NEB Inc., Ipswich, MA, USA), with 1 µg of total RNA. The library sequencing was performed on a HiSeq 2000 sequencing platform, with the 150-bp paired-end reads strategy (Novogene Bioinformatics Technology Co. Ltd., Tianjing, China).

To identify the genes involved in pheromone production, we assembled clean reads of the PGs and 12 other tissues previously sequenced in *A. yunnanensis* (Li et al., 2021), using *de novo* Trinity (version 2.5.1) (Grabherr et al., 2011). After assembling, the clustering analysis with Corset v1.05 was conducted (Davidson & Oshlack, 2014), and further the longest transcript was selected as one unigene. The combination of all the unigenes was defined as the unigene transcriptome. Gene expression in each tissue was determined following the procedures: (1) using Bowtie2 to align clean reads of each sample against the unigene transcriptome (Langmead & Salzberg, 2012); and (2) using RSEM v1.2.15 (Li & Dewey, 2011) to calculate FPKM (fragments per kilobase of transcript per million mapped reads) values (Trapnell et al., 2010).

### Identification of candidate genes involved in the pheromone biosynthesis

We employed TBLASTN to identify the genes associated with the sex pheromone production from the transcriptome. The protein sequences of 10 specific gene families were collected and pooled as queries (*i.e.*, ACCs, FASs, FADs, FARs, aldehyde reductases (ARs), aldehyde dehydrogenases (ALDHs), AOs/ADHs, ATFs, fatty acid transport proteins (FATPs) and AOXs), from *Noorda blitealis* (Zhang et al., 2021), two *Spodoptera* species (*S. exigua* and *S. litura*) (Zhang et al., 2015; Zhang et al., 2017), three *Helicoverpa* species (*H. armigera*, *H. assulta* and *H. zea*) (Li et al., 2015; Dou et al., 2019a) and *Plutella xylostella* (He et al., 2017). For the identification of AOXs, except for the above six moths, the corresponding protein sequences from three additional moths, *Amyelois transitella*, *Chilo suppressalis* and *Ostrinia furnacalis*, were included (Zhang et al., 2021). To avoid missing any pheromone biosynthesis and degradation related genes, newly identified genes were used as queries to screen the transcriptome. If two or more genes shared above 95% identities at the amino acid level, based on sequence characteristics and the similarity with those in other species, only one sequence was retained. Short fragments with below 80 amino acids were discarded.

### Phylogenetic analysis

Multiple alignments of amino acid sequences were conducted using MAFFT v7.450 with the parameters (E–INS–I algorithm, scoring matrix of BLOSUM62, gap open penalty of 1.53 and offset value of 0.123) (Katoh & Standley, 2013). Based on the aligned protein sequences, we inferred the maximum-likelihood phylogenetic tree of each gene family using FastTree v2.1.12, under the Whelan and Goldman (WAG) model with 1,000 replicates (Price, Dehal & Arkin, 2010). In brief, the FAD data set comprised 112 sequences from 26 moth species, including 22 relatives in *A. yunnanensis*. In the FAR data set, we selected 119 proteins from 35 insects, with 24 AyunFARs. In the AOX tree, 58 AOX sequences were used, along with 20 xanthine dehydrogenase (XDH) proteins from 10 lepidopteran species as the outgroup.

### RNA isolation and cDNA synthesis

Total RNAs of larval and adult tissues were isolated using TRIzol reagent (Ambion, Life Technologies, Carlsbad, CA, USA), along with the protocol. To avoid the contamination, a gDNA Eraser was used to eliminate gDNA from purified RNA samples. Next, the PrimeScript RT reagent Kit (TaKaRa, Dalian, Liaoning, China) was used to synthesize cDNA templates following the manufacturer’s suggestions. In particular, an equal amount of total RNA from five tissues, viz. heads without antennae, thoraxes, abdomens without PGs, legs and wings, was mixed as bodies (up to 1 µg).

### Expression profiling analysis of candidate genes in tissues

Based on the FPKM values obtained by transcriptome sequencing, we first built an expression profile map of genes in 13 tissues, including the PGs. Data with FPKM values were presented using heatmaps generated by GraphPad Prism 7.00 (GraphPad Software Inc., San Diego, CA, USA). Next, we employed reverse transcription PCR (RT–PCR) to examine the expression of genes in larval and adult tissues. Each reaction contained a total volume of 25 µL mixture with 2 µL of cDNA, 2.5 µL of 10 × PCR Buffer (Mg^2+^ plus), 2 µL of dNTP Mixture (each 2.5 mM), each 1.5 µL of forward and reverse primers (10 µM), 0.15 µL of Taq DNA Polymerase (5 U/µL) (TaKaRa, Dalian, Liaoning, China) and 15.35 µL of sterile water. The reaction procedures were 3 min at 94 °C, followed by 35 cycles of 30 s at 94 °C, 30 s at 58 °C and 40 s at 72 °C, and a final extension of 5 min at 72 °C. For RT–PCR analyses of genes, negative controls using sterile water as the template were carried out. If the expression of one gene with RT–PCR was inconsistent with FPKM results, at least two biological replicates were performed (Supplemental Information 6). Gene specific primers were designed by Primer Premier 5.0 (PREMIER Biosoft International, CA, USA) (Table S1). An internal reference gene, ribosomal protein L8 (*AyunRPL8*) (Li et al., 2021), was used as the quality and quantity control of cDNA templates. Uncropped gel pictures of all selected genes were seen in Supplemental Information 6.

According to the FPKM and RT–PCR results, we further selected 23 genes showing high expression in the PGs to determine their relative expression ratios in PGs and other tissues by quantitative real-time PCR (qPCR). Each reaction mixture consisted of 2 µL of cDNA, 10 µL 2 × SybrGreen qPCR mastermix (DBI^^®^^ Bioscience, Germany), each 0.8 µL of forward and reverse primers (10 µM), and 6.4 µL of sterile water. The reactions were run on three independent biological templates, each with three technical replicates. qPCR were conducted on a qTOWER 2.2 instrument (Analytic Jena AG, Germany), with the cycling conditions: 2 min at 95 °C, followed by 40 cycles of 10 s at 95 °C, 31 s at 58 ° C and 30 s at 72 °C. For each gene, fluorescence signals were collected at the elongation step of 72 ° C. To verify the specificity of the amplification products, we analyzed the melting curves of each gene. The primers of genes were designed using Beacon Designer 8.14 (PREMIER Biosoft International, CA, USA) (Table S1). The cycle threshold (CT) values of target and reference genes were seen in Supplemental Information 7. Relative expression levels of candidate genes were computed by using an internal reference, ribosomal protein S4 (*AyunRPS4*) (Li et al., 2021), to normalize the expression of target genes with a Q-GENE method (Muller et al., 2002; Simon, 2003). Raw data of relative expression levels of target genes were seen in Supplemental Information 8.

To summarize the numbers of genes in tissues of *A. yunnanensis*, one gene was regarded to have the expression in one tissue as the following standards: (1) the FPKM values > 1, or (2) RT–PCR or qPCR evidence.

### Statistical analysis

To compare qPCR data of genes among different tissues, we performed a one–way analysis of variance (ANOVA) and a multiple comparison test (Fisher ′s least significant difference, LSD), implemented in IBM SPSS Statistics 21.0 (SPSS Inc., Chicago, IL, USA). Significant difference among the expression data was set at *P* <0.05.

## Results

### Transcriptome sequencing and analysis of the *A. yunnanensis* PGs

After cleaning the sclerotized cuticles of the *A. yunnanensis* PGs, we constructed and sequenced its RNA–Seq library through Illumina sequencing (Fig. 1). Totally, 47,766,846 raw reads of the PG tissues were generated. After removing the redundant and low quality sequences, the remaining 47,632,610 reads were set as clean reads [up to 7.14 gigabases (G)], and accounted for 99.72% of the initial sequences. Coupled with clean data of 12 other tissues in a previous study (Li et al., 2021), the Trinity and Corset analyses resulted in the generation of 54,297 unigenes, approximately 38.42% of which (20,859 genes) showed detectable expression levels in the PGs (FPKM >1). Besides, 5746 of the genes had relatively high expression with the FPKM values of above 10, and 683 for high expression (FPKM >100) in the PGs (Table 2).

**Table 2:** Transcriptome sequencing statistics of the PGs in *A. yunnanensis*.

Sample	PG
Raw read	47,766,846
Clean read	47,632,610
Size (Gb)	7.14
Error (%)	0.02
Q20 (%)	96.57
Q30 (%)	91.10
GC content (%)	44.69
Containing N (%)	0.00
Low quality (%)	0.28
Adapter related (%)	0.00
Unigene (FPKM > 0)	54,297
Unigene (FPKM > 1)	20,859
Unigene (FPKM > 10)	5746
Unigene (FPKM > 100)	683
Unigene (FPKM > 1000)	78

Focusing on the 78 highly expressed genes in the PGs (FPKM >1000), it was noticed that the majority of unigenes showed high sequence similarities to ribosomal proteins in the National Center for Biotechnology Information (NCBI) Non-redundant (NR) protein sequence database, representing the most abundant presence of c80733_g1_i0 (FPKM = 6934.78) that was homologous to a 60S ribosomal protein L39 in *Odontomachus brunneus* (accession number: XP_032686856.1). Other putative highly expressed genes included seven heat shock proteins (HSPs: c78225_g0_i0, c83758_g0_i0, c44035_g0_i0, c61844_g0_i0, c82375_g1_i1, c58568_g1_i1 and c54533_g0_i0), four chemosensory proteins (CSPs: c66899_g2_i0, c79250_g0_i0, c81378_g0_i0 and c60713_g1_i1), two cuticle proteins (CPs: c63479_g0_i0 and c41287_g1_i0), one carboxylesterase (COE: c35888_g822_i0) and one vitellogenin (c31663_g0_i0). In addition to those, several house-keeping genes were found to have particularly high expression in the PGs, including actin (c55402_g1_i0), elongation factor (c69234_g0_i1), glyceraldehyde-3-phosphate dehydrogenase (c35888_g1343_i0), cytochrome c oxidase subunit I (c62622_g1_i0) and III (c50440_g13_i0). However, eight unigenes had no homology to sequences in the GenBank NR protein database (Table S2).

In comparison to the genes expressed in 12 other tissues, there were 174 detectable relatives exclusively transcribed in the PGs (1 < FPKM < 60). Among them, virtually all the genes had short nucleotide sequences (<800 bp), except for c26231_g0_i0 (1864 bp). Further, BLASTX results revealed that many of the genes had no significant hits to sequences in the GenBank database, with the exception of 19 genes that were similar to insect proteins (Table S3).

### Gene comparison

The PG of the female moth is a specialized tissue and has been extensively characterized with respect to transcriptomics, gene identification and gene function (Ma & Ramaswamy, 2003; Jurenka, 2021). In the present study, we compared the numbers of genes associated with the sex pheromone production, representing nine pheromone biosynthesis enzyme families and one pheromone degradation enzyme family from 24 moth species of nine families. Of these species, *A. yunnanensis* possessed the largest number of 191 genes, followed by *N. blitealis* (114), *Ephestia cautella* (102) and *P. xylostella* (99). The remaining species varied from 14 in *Grapholita molesta* and *Grapholita dimorpha* to 77 in *Antheraea pernyi*. In each gene family, most species had one ACC and four FATPs. In comparison, the numbers of other gene repertoires were variable, such as FADs (four to 22), FARs (one to 28), AOs/ADHs (five to 34) and ATFs (one to 63). Among the 24 species studied, the AOX gene family of nine moth species was identified from the PG transcriptomes, showing variable and small numbers of genes (1–7 relatives) (Table 1). The nucleotide and amino acid sequences of all the 191 genes in *A. yunnanensis* were listed in Supplemental Informations 9 and 10, respectively.

### Candidate genes involved in the pheromone production

### Acetyl-CoA carboxylase and fatty acid synthase

As the initial step of the sex pheromone biosynthesis, both ACC and FAS are responsible for the synthesis of saturated fatty acid precursors (Volpe & Vagelos, 1973). Here, we identified 15 transcripts encoding three ACCs and 12 FASs from the transcriptome. Six of the genes were full-length sequences, including two *ACCs* (*AyunACC1*–*2*) and four *FASs* (*AyunFAS1*–*4*). *AyunACC1* and *ACC2* encoded 2355 and 704 amino acids, respectively. Four full-length FAS transcripts shared a similar sequence length to each other, ranging from 2294 amino acids in AyunFAS1 to 2421 in AyunFAS3. Other nine genes were partial sequences, including one *ACC* (*AyunACC3*) and eight *FASs* (*AyunFAS5*–*12*) (Table S4).

### Fatty acyl desaturase

In the sex pheromone biosynthesis of female moths, double bonds of unsaturated fatty acids were introduced by FADs (Fujii et al., 2015; Xia et al., 2019). In *A. yunnanensis*, 22 transcripts were identified and shared high sequence identities to FADs in lepidopteran species (>55% amino acid identities). Of the 22 *FAD* genes, 14 relatives were predicted to have complete open reading frames (ORFs) where the shortest gene was *AyunFAD5* encoding 331 amino acids and the longest FAD for *AyunFAD7* (452 amino acids). Other eight transcripts were fragments with various sequence lengths that ranged from 176 amino acids in AyunFAD22 to 409 in AyunFAD19 (Table S4).

**Figure 2:**
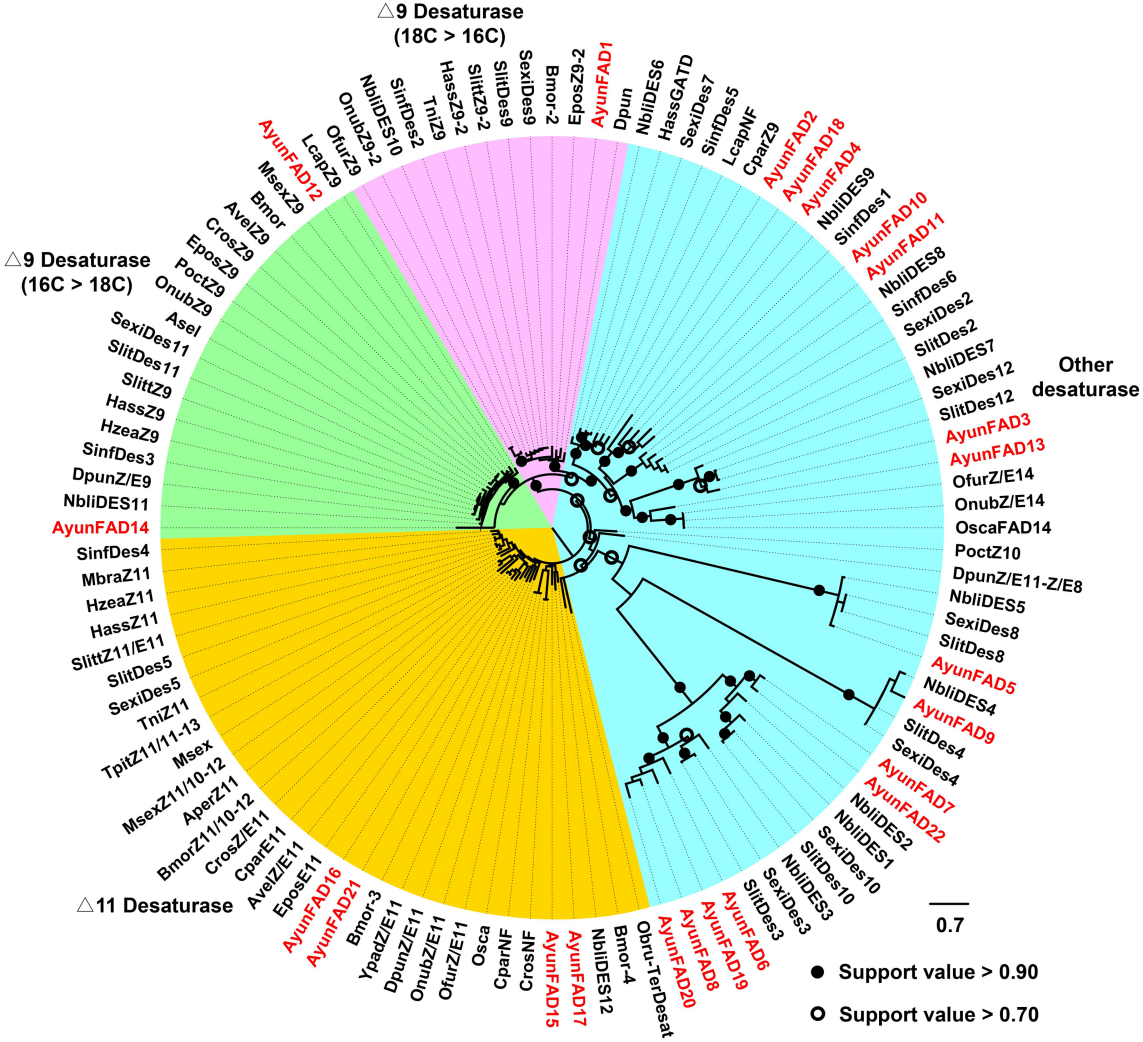
Maximum-likelihood phylogenetic analysis of fatty acyl desaturases (FADs) in *A. yunnanensis* and 25 other moths. Candidate AyunFADs newly identified in this study were presented in red. Based on putative functional roles of FADs in moths, 22 AyunFADs were divided into four subfamilies, with the majority of them clustering into the other desaturase clade. Asel, *Ascotis selenaria cretacea*; Avel, *Argyrotaenia velutinana*; Ayun, *Achelura yunnanensis*; Bmor, *Bombyx mori*; Cpar, *Choristoneura parallela*; Cros, *Choristoneura rosaceana*; Dpun, *Dendrolimus punctatus*; Epos, *Epiphyas postvittana*; Lcap, *Lampronia capitella*; Mbra, *Mamestra brassicae*; Msex, *Manduca sexta*; Nbli, *Noorda blitealis*; Sexi, *Spodoptera exigua*; Sinf, *Sesamia inferens*; Slit, *Spodoptera litura*; Slitt, *Spodoptera littoralis*; Tpit, *Thaumetopoea pityocampa*; Ofur, *Ostrinia furnacalis*; Obru, *Operophtera brumata*; Onub, *Ostrinia nubilalis*; Osca, *Ostrinia scapulalis*; Poct, *Planotortrix octo*; Hass, *Helicoverpa assulta*; Hzea, *Helicoverpa zea*; Ypad, *Yponomeuta padellus* and Tni, *Trichoplusia ni*.

In the phylogenetic analysis of FADs from 26 moths, 22 AyunFADs in *A. yunnanensis* were distributed in four clades, with the majority of members in other desaturases (15 relatives). In two conserved clades of △9 desaturases (16C >18C and 18C >16C), three members of FADs (AyunFAD1, FAD12 and FAD14) shared over 80% amino acid identities with FADs in other lepidopteran species. The △11 desaturases comprised four members of AyunFADs, with an average of 71.22% amino acid identity among them (Fig. 2).

### Fatty acyl-CoA reductase, alcohol oxidase/dehydrogenase and acetyltransferase

In the formation of functional groups of the Type I sex pheromones (alcohols, aldehydes and acetate esters), three enzyme families of FARs, AOs/ADHs and ATFs are essential (Jurenka, 2004). From the transcriptome, we detected a total of 121 transcripts homologous to FAR, AO/ADH or ATF sequences in other lepidopteran species. Among them, the majority of the genes (19 of 24 *FARs*, 32 of 34 *AOs/ADHs* and 59 of 63 *ATFs*) were full-length sequences. A blast search of the proteins against the NCBI NR protein sequence database indicated that most of the genes, especially for *AOs/ADHs* and *FARs*, showed high sequence identities with those in other lepidopteran species (>55%) (Table S4).

**Figure 3:**
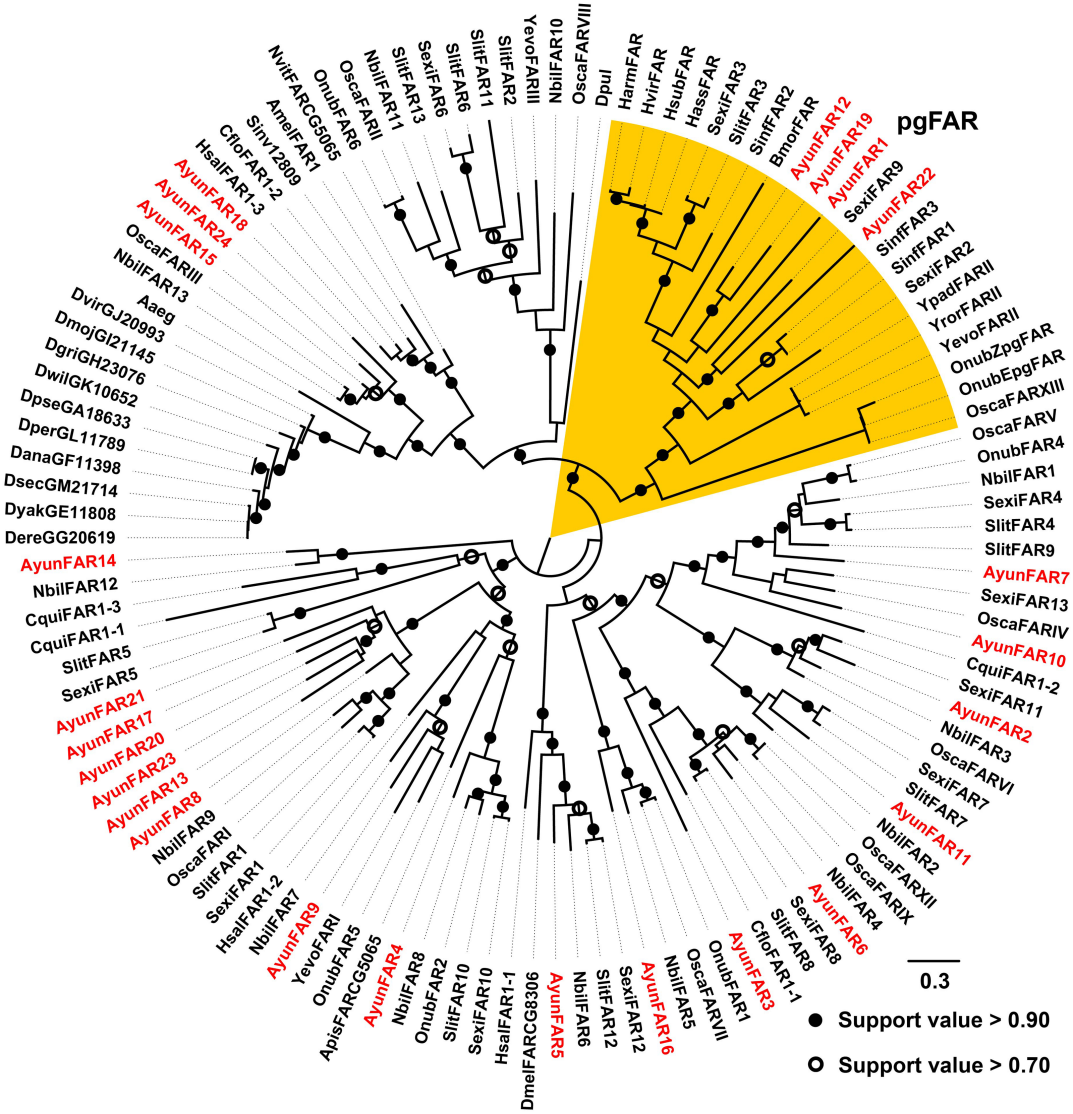
Maximum-likelihood phylogenetic analysis of fatty acyl-CoA reductases (FARs) in 35 insects. A PG-specific group of FARs in Lepidoptera, namely pgFAR, was shaded in orange. Candidate AyunFARs newly identified in this study were presented in red. Aaeg, *Aedes aegypti*; Amel, *Apis mellifera*; Apis, *Acyrthosiphon pisum*; Ayun, *Achelura yunnanensis*; Cflo, *Camponotus floridanus*; Cqui, *Culex quinquefasciatu*; Dana, *Drosophila ananassae*; Dere, *Drosophila erecta*; Dgri, *Drosophila grimshawi*; Dmel, *Drosophila melanogaster*; Dmoj, *Drosophila mojavensis*; Dpul, *Daphnia pulex*; Dvir, *Drosophila virilis*; Dyak, *Drosophila yakuba*; Dper, *Drosophila persimilis*; Dpse, *Drosophila pseudoobscura*; Dsec, *Drosophila sechellia*; Dwil, *Drosophila willistoni*; Hsal, *Harpegnathos saltator*; Nvit, *Nasonia vitripennis*; Sinv, *Solenopsis invicta*; Yevo, *Yponomeuta evonymellus* and Yror, *Yponomeuta rorrellus*. The abbreviations of other species are seen in Fig. 2.

In previous studies, a PG-specific group of the Lepidoptera is proposed as pgFAR (Moto et al., 2003; Hagstrom et al., 2012). Our current study identified four members in this clade (AyunFAR1, FAR12, FAR19 and FAR22), but each of which did not share high amino acid identities with FARs in other lepidopterans (<55%). The remaining 20 AyunFARs grouped into various clades, in which five relatives (AyunFAR13, FAR17, FAR20, FAR21 and FAR23) formed a monophylogenetic cluster (Fig. 3).

### Other enzymes in the pheromone biosynthetic pathways

Except for the six gene families described above, here we additionally identified 10 *ARs*, 13 *ALDHs* and four *FATPs*, with 25 full-length relatives. Ten AyunARs exhibited high sequence identities (>58%) with reductases in other lepidopterans. All the AyunALDHs, except for AyunALDH2 and ALDH9, had above 60% amino acid identities with the proteins in other lepidopteran species. Four sequences in *A. yunnanensis* homologous to FATPs shared high conservation among lepidopterans (71.45–85.07% amino acid identities) (Table S4).

### Candidate genes involved in the pheromone degradation

Moth AOXs are capable of degrading aldehydes into carboxylic acids, and thus respond to aldehyde-related components in the degradation pathways of sex pheromones (Choo et al., 2013). In *A. yunnanensis*, six genes encoding AOXs were found with two copies in the AOX6 group. However, the ortholog of AOX1 was absent in the transcriptome. All these genes had full-length sequences encoding 1204–1301 amino acids. They shared a moderate amino acid identity to AOXs in other moths, ranging from 58.58% to 68.62% identities. In addition, two candidate XDH genes were identified from the transcriptome, with 1339 amino acids in AyunXDH1 and 1352 in AyunXDH2. Of the two genes, AyunXDH1 had a high amino acid identity (80.81%) with the XDH protein in *Manduca sexta* (accession number: XP_030020628.1) (Table S4).

**Figure 4:**
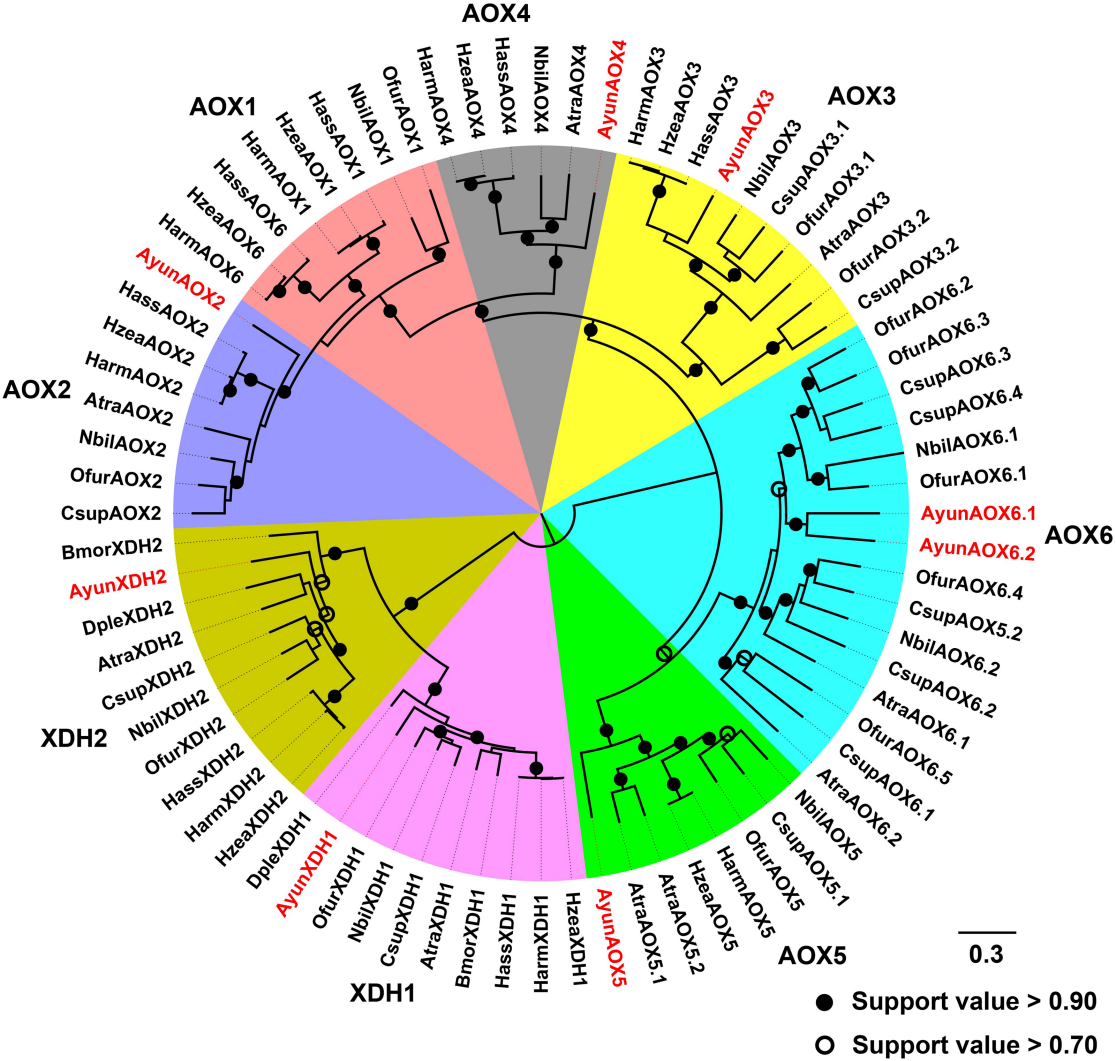
Maximum-likelihood phylogenetic analysis of aldehyde oxidases (AOXs) in *A. yunnanensis* and seven other moths, together with 20 XDH proteins used as the outgroup. Following this classification system of AOXs previously described in Lepidoptera (Zhang et al., 2021), six AyunAOXs clustered into six subfamilies, in which AyunAOX6 harbored two relatives. Candidate AyunAOXs and XDHs newly identified in this study were presented in red. Atra, *Amyelois transitella*; Csup, *Chilo suppressalis* and Dple, *Danaus plexippus*. The abbreviations of other species are seen in Fig. 2.

Based on a previous classification system of AOXs in Lepidoptera (Zhang et al., 2021), AyunAOXs clustered in five orthologous clades: AOX2–AOX6. Members of an AOX2 clade functioned in the degradation of aldehyde compounds contained AyunAOX2, which shared an average of 66.87% identity with other members in this clade (Fig. 4).

### Expression profile of the pheromone biosynthesis and degradation genes determined by RNA–Seq

Combining the sequencing data of PGs and 12 other tissues, we first calculated the expression levels of all the genes in tissues as measured by FPKM. Ten transcripts (*AyunACC3*, *ATF41*, *ATF53*, *ATF61*, *ALDH12*, *AO9*, *AO31*, *AO33*, *FAD23* and *FAS12*) were not found in the unigene transcriptome, but presented in the transcript transcriptome. Out of the 181 identified genes, 128 relatives had detectable transcription in the *A. yunnanensis* PGs (FPKM >1). Except for the PG tissues, most of the genes were also transcribed widely in other tissues. Three transcripts displayed the most abundant expression in the PGs, *i.e., AyunALDH8* (FPKM: 180.99), *ATF5* (FPKM: 180.08) and *ATF12* (FPKM: 187.92). Compared to other tissues, seven genes (*AyunATF12*, *ATF54*, *ATF58*, *FAD7*, *FAD22*, *FAR10* and *FAR11*) were enriched in the PGs of *A. yunnanensis* with at least 2-fold higher expression. In particular, *AyunATF54* transcriptional levels had over 11-fold difference between PGs and other tissues (Supplemental Information 11 and Fig. S1).

Focusing on putative roles of the 181 genes in olfaction excluding 10 relatives having no FPKM values, we detected their expression in female and/or male antennae. Similar to the number of the PG-expressed genes, totally 127 relatives were found in the antennae as their FPKM values were above 1. *AyunALDH1* and *ALDH8* were the most abundantly expressed genes in female (FPKM: 281.71 and 426.95, respectively) and male (FPKM: 301.46 and 408.74, respectively) antennae. Notably, some genes were enriched in the antennae with at least 2-fold higher transcription compared to that in other tissues. *AyunAOX2* displayed a particularly high expression in the antennae (15-fold difference relative to other tissues). Apart from those, *AyunALDH10*, *FAR14* and *AOX3* had relatively high expression abundance in the antennae, with over 5-fold difference compared to other tissues (Supplemental Information 11 and Fig. S1).

### Expression profile of the pheromone biosynthesis and degradation genes determined by PCR

**Figure 5:**
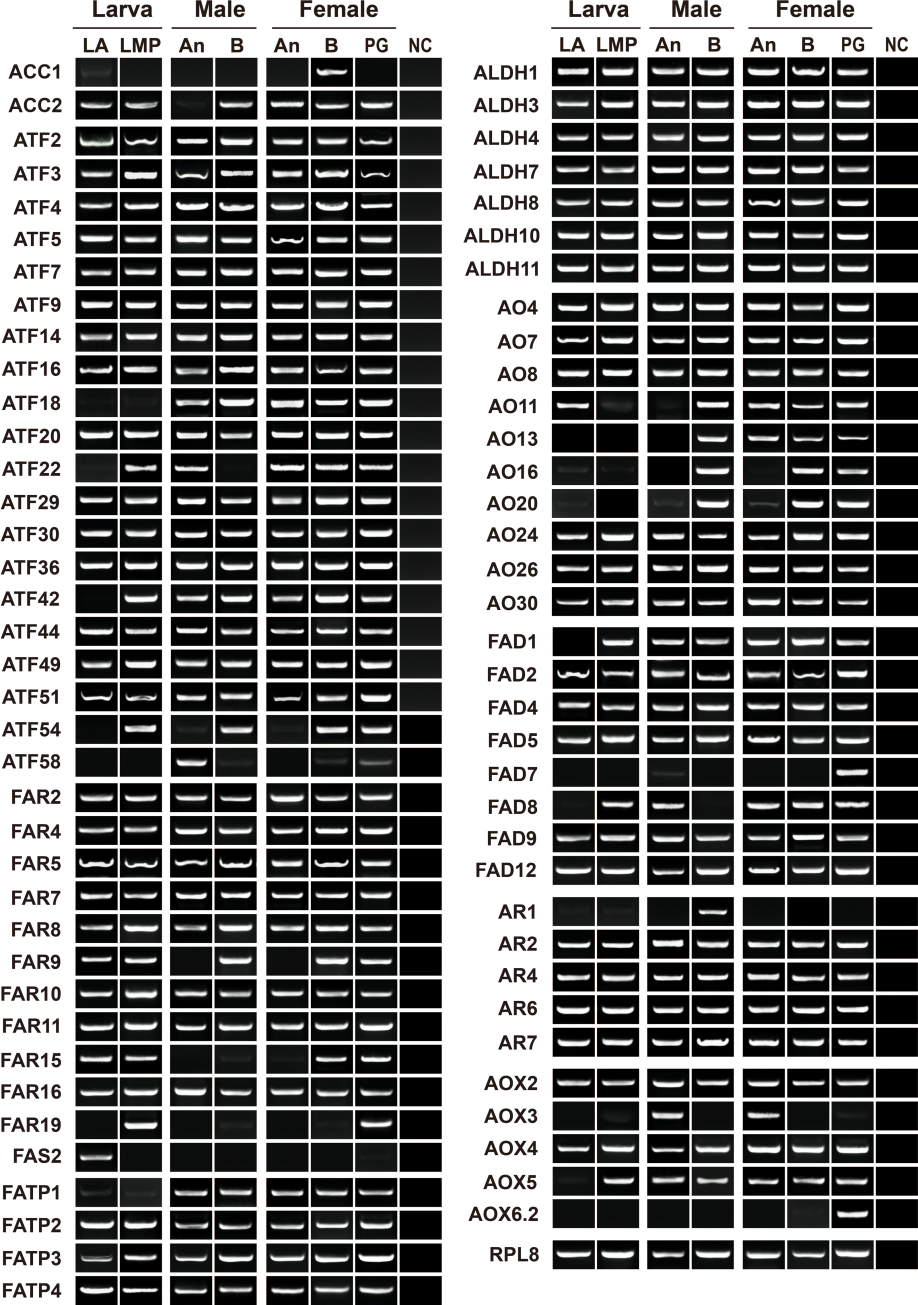
RT-PCR analyses showing the expression profiles of 73 pheromone biosynthesis related genes in larval and adult tissues. The quality of cDNA templates was checked by a reference gene, *AyunRPL8*. LA, larval antennae; LMP, larval maxillary palps; An, adult antennae, PG, pheromone glands and B, mixed adult bodies without antennae and pheromone glands.

To validate and reconstruct the transcripts, we randomly selected 74 relatives, including two *ACCs*, one *FAS*, eight *FADs*, five *ARs*, 11 *FARs*, 20 *ATFs*, seven *ALDHs*, 10 *AOs*, four *FATPs* and six *AOXs*. Except for *AyunAOX6.1*, all the genes were presented in at least one tissue, over 90% of the genes in three or more tissues, and approximately 68.49% of the genes in all tested tissues similar to the expression profile of a house-keeping gene, *AyunRPL8*. Four genes, *AyunACC1*, *FAS2*, *AR1* and *AOX3*, were specifically expressed in female bodies, larval antennae, male bodies and adult antennae, respectively, but absent in the PGs. In contrast, *AyunFAD7* and *AOX6.2* appeared to be PG-specific transcripts. Intriguingly, a number of genes were presented in larval (55 relatives) and adult (62 relatives) antennae, as well as larval maxillary palps (60 relatives). In female antennae, some genes were preferentially expressed, including *AyunACC2*, *AO11* and *AO13* (Fig. 5).

**Figure 6:**
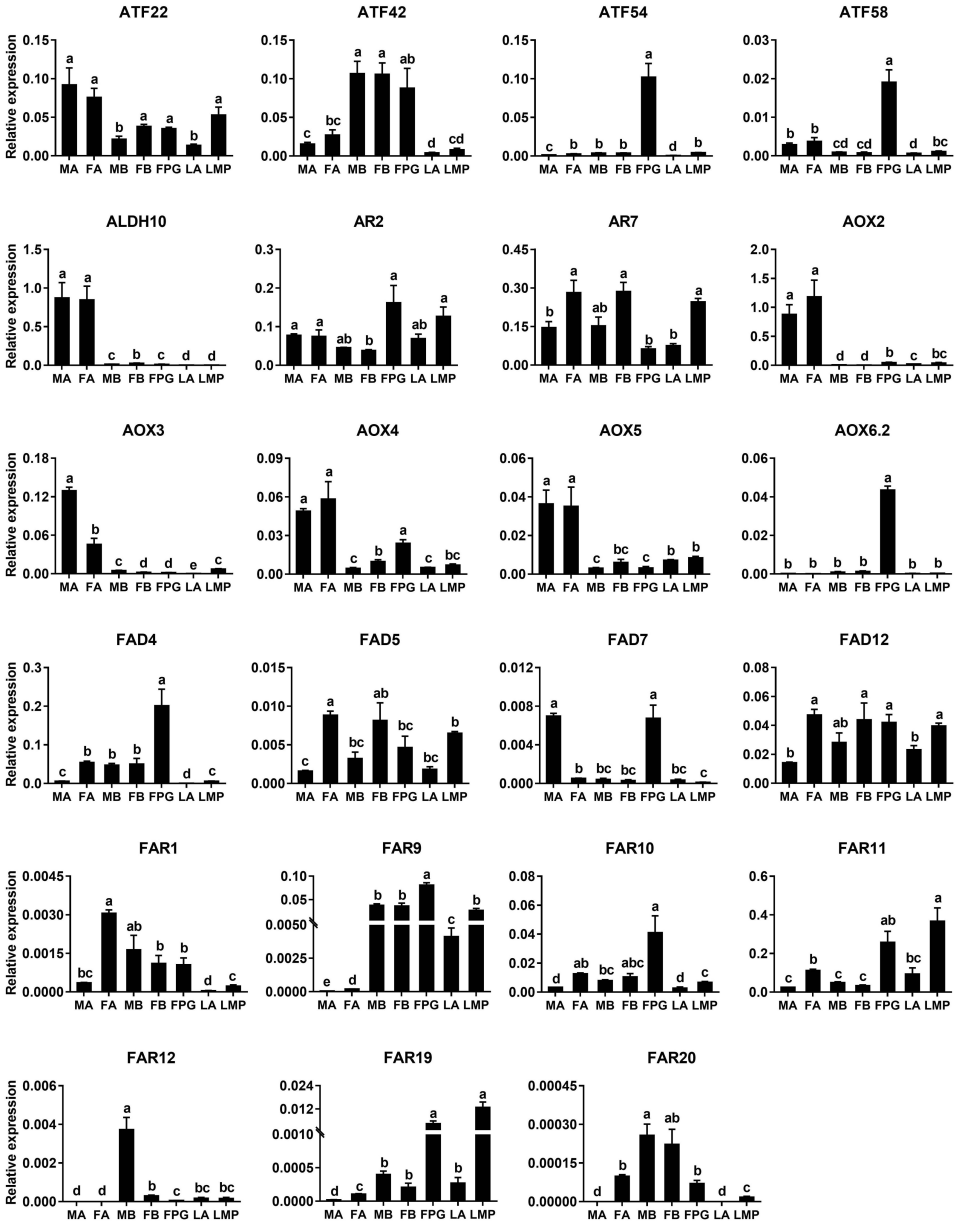
qPCR analyses showing the relative expression of 23 pheromone biosynthesis related genes in larval and adult tissues. The expression levels of each gene in various tissues were computed relative to a reference gene, *AyunRPS4*. Data are plotted with mean ± SEM. LA, larval antennae; LMP, larval maxillary palps; MA, male antennae; MB, mixed male bodies without MA and PGs; FA, female antennae; FB, mixed female bodies without FA and PGs, and FPG, female pheromone glands.

Based on the results of RNA–Seq and RT–PCR, qPCR was further employed to examine relative expression levels of 23 genes in antennae, PGs and bodies. Of the four *ATF* genes, *AyunATF22* and *ATF42* were broadly presented in various tissues, whereas *AyunATF54* and *ATF58* had a significantly higher expression in the PGs than other tissues. A similar result about the significantly PG-enriched presence was observed in *AyunAOX6.2*, *FAD4* and *FAR9*. Most of the remaining genes displayed a wide tissue expression, such as *AyunAR2*, *AR7*, *AOX4*, *AOX5*, *FAD5*, *FAD12*, *FAR1*, *FAR10* and *FAR11*. Moreover, it was observed that the expression of eight genes was significantly female-biased (*AyunAR7*, *FAD4*, *FAD5*, *FAD12*, *FAR1*, *FAR10*, *FAR11* and *FAR20*), and two for male-biased expression (*AyunAOX3* and *FAD7*) (Fig. 6).

## Discussion

Like most other moths, the zygaenid species utilize a specialized tissue, the PG, to produce the sex pheromones that are attractive to males for mating (Percy-Cunningham & Macdonald, 1987; Raina et al., 2000; Allison & Cardé, 2016). Up to now, over 1000 species in the Zygaenidae family have been described (Mitter, Davis & Cummings, 2017), but the sex pheromones of only three zygaenid moths are identified, representing the Procridinae subfamily of *Harrisina brillians* (Myerson, Haddon & Soderstrom, 1982), *Illiberis rotundata* (Subchev et al., 2009) and *Theresimima ampellophaga* (Subchev et al., 1998). Apart from that, sex attractants of 58 zygaenid species have been reported, but not *A. yunnanensis* (Sayed, 2021). Moreover, the genes associated with the pheromone biosynthesis and degradation in Zygaenidae remain to be identified and poorly characterized. In the current study, we concentrated on a serious defoliator of the Rosaceae family, *A. yunnanensis* (Li et al., 2020; Li et al., 2021), and characterized its PG transcriptome and the pheromone production related genes. To the best of our knowledge, this is the first survey for the gene repertoires responsible for the biosynthesis and degradation of sex pheromones in Zygaenidae.

With a particular focus on the highly expressed genes in the PGs, it was noticed that the majority of the most abundant transcripts were similar among moths, including a reproductive-related protein (vitellogenin), CSPs possibly involving sex pheromone releasing, HSPs and house-keeping genes (Valle, 1993; Gu et al., 2013; Antony et al., 2015; Zhang et al., 2015; Cha et al., 2017; Pelosi et al., 2018). The common presence of some proteins like CSPs, HSPs and vitellogenins implied their key roles in the PGs, though the physiological functions remained to be unraveled. In comparison to other tissues, most of the PG-specific genes in *A. yunnanensis* (approximately 89.08%) had short sequences that showed no similarity to protein sequences in the NCBI NR protein sequence database. Such results were likely to reflect that some PG-exclusive genes in Lepidoptera were not identified or incomplete annotation of the PG-expressed genes in the GenBank database, as indicated in *Agrotis segetum* (Strandh et al., 2008), *Heliothis virescens* (Vogel et al., 2010), *Agrotis ipsilon* (Gu et al., 2015) and *E. cautella* (Antony et al., 2015).

In the comparison of gene numbers, there were variable genes in each gene family, though they were retrieved exclusively from the PG transcriptomes. As one of the differences of gene numbers, it may be attributed to the sequencing depth of the PG transcriptomes, such as *A. yunnanensis* (47,632,610 clean reads), *E. cautella* (231, 851, 937 clean reads) (Antony et al., 2015), *S. litura* (63,209,172 clean reads) (Zhang et al., 2015), *Maruca vitrata* (119,454,916 clean reads) (Cha et al., 2017) and *A. segetum* (53,235,252 clean reads) (Ding & Lofstedt, 2015). In addition to that, the transcriptome sequencing and assembly of multi-tissues also facilitated the identification of the pheromone biosynthesis and degradation genes in the PGs, further supported by a broad tissue distribution of the pheromone biosynthesis genes in *A. yunnanensis* and other moth species (Zhang et al., 2014b; Xia et al., 2015; Yang, Tao & Zong, 2020). Except for the possible reasons mentioned above, we cannot exclude other possibilities such as the difference of sex pheromone components among species.

In moths, AOXs could be distinguished into six groups with a recently proposed clade of AOX6 in pyralid species (Zhang et al., 2021). Based on the phylogenetic analysis of lepidopteran AOXs, it was found that the *A. yunnanensis* transcriptome did not comprise a member homologous to AOX1. As indicated in a previous study, two species in the Pyralidae family, *A. transitella* and *C. suppressalis*, also lost the orthologs of AOX1 (Zhang et al., 2021). In addition to that, to check the existence of the AOX1 ortholog in Zygaenidae, we downloaded the only available genome assembly in *Zygaena filipendulae*, from public databases (accession number: GCA_907165275.1, version ilZygFili1.1). Unfortunately, a TBLASTN search did not found an orthologous AOX1. Considering the absence of AOX1 in *Z. filipendulae* and some moth species (Zhang et al., 2021), we postulated that *A. yunnanensis* may lose the AOX1 gene.

As evidenced in functional experiments, antennae-enriched AOX genes in *M. sexta* (Rybczynski, Reagan & Lerner, 1989), *A. transitella* (Choo et al., 2013) and *P. xylostella* (Wang et al., 2021) clustering in the AOX2 clade were responsive to aldehyde-related compounds. Here, AyunAOX2 had high conservation with other AOX2 members in moths, suggestive of its putative roles in degrading aldehyde compounds, further supported by the observation that this gene was significantly abundant in the antennae. In other AOX groups, although the roles of these genes (*e.g.*, *AyunAOX3*, *AOX4* and *AOX5*) have not been characterized, their highly expressed characteristics in the antennae suggested the involvement in the degradation of aldehyde compounds (Pelletier et al., 2007; Zhang et al., 2014b; He et al., 2017). In particular, *AyunAOX3* with a significantly male-biased expression may be involved in the degradation of aldehyde sex pheromones.

In the expression profiles of genes, although most of the genes were detected in the PGs of *A. yunnanensis*, the PG-exclusive or enriched genes had a relatively small number. This may be related to transcriptome-wide sequencing as well as an extensive tissue-, stage- and sex- expression profile of the genes, as the previous studies mainly sequenced the PG transcriptomes and investigated the expression profiles of genes in PGs and bodies without PGs (Gu et al., 2013; Zhang et al., 2017; Wang et al., 2020; Zhang et al., 2021). Nevertheless, some significantly PG-abundant genes were obtained in the PGs of *A. yunnanensis* (*AyunATF54*, *ATF58*, *FAD4*, *FAD7*, *FAR9* and *FAR10*), suggesting that they were strong candidates for the biosynthesis of sex pheromones in this species. Focusing on two PG-biased desaturases of *AyunFAD4* and *FAD7* clustering in the other desaturases, it was found that this expression pattern was inconsistent with the results of most moths (Zhang et al., 2014b; Xia et al., 2015; He et al., 2017), but similar to those of *SlitDES2* and *DES10* in *S. litura* (Zhang et al., 2015), as well as *SexiDES2* and *DES10* in *S. exigua* (Zhang et al., 2017). Out of the 24 *AyunFARs*, four members that clustered in the pgFAR clade did not show a PG-dominant expression, inconsistent with the results observed in most moth species (Moto et al., 2003; Zhang et al., 2015; He et al., 2017; Zhang et al., 2017), but similar to that of *SinfFAR1* and *FAR3* genes in *Sesamia inferens* (Zhang et al., 2014b).

## Conclusions

In summary, our current study generated a large number of clean reads (7.14 G) from the PG tissues of *A. yunnanensis*. Transcriptome-wide analysis combined with RNA–Seq data derived from 12 other tissues led to the identification of 191 genes associated with the biosynthesis and degradation of sex pheromones. Out of the 191 genes, 128 relatives were detected in the PGs and 127 in the antennae with FPKM (>1) and PCR supports. In addition, we explored a wide tissue- and sex- distribution of nearly all the genes, and characterized phylogenetic relationships of three enzyme gene families (FADs, FARs and AOXs) in *A. yunnanensis* and other insects. Except for the PG-enriched genes, some relatives were transcribed exclusively in the antennae, several of which showed a sex-biased expression. Together, our study surveys, for the first time, a global resource for exploring putative roles of the pheromone production related genes in a zygaenid moth, and identifies candidate genes involved in the pheromone biosynthesis and degradation as well as olfactory behaviors.

## Supplemental Information

10.7717/peerj.12641/supp-1 Supplemental Information 1 The heatmaps showing the expression profiles of 181 pheromone biosynthesis related genes in different tissues of both sexes from *A. yunnanensis* Different colors denote the expression abundance of genes in tissues as measured by FPKM values.

10.7717/peerj.12641/supp-2 Supplemental Information 2 Primers used for the expression profiles of the pheromone production related genes in *A. yunnanensis*

10.7717/peerj.12641/supp-3 Supplemental Information 3 The most highly expressed genes in the *A. yunnanensis* PG (FPKM >1000)

10.7717/peerj.12641/supp-4 Supplemental Information 4 The specifically expressed genes in the *A. yunnanensis* PG

10.7717/peerj.12641/supp-5 Supplemental Information 5 Candidate genes involved in the biosynthetic pathway of sex pheromones in *A. yunnanensis*

10.7717/peerj.12641/supp-6 Supplemental Information 6 Raw gel images of 74 pheromone biosynthesis and degradation related genes and *AyunRPL8* from *A. yunnanensis* in RT-PCR analyses

10.7717/peerj.12641/supp-7 Supplemental Information 7 Cycle threshold (CT) values of 23 pheromone biosynthesis and degradation related genes and *AyunRPS4* from *A. yunnanensis* in qPCR analyses

10.7717/peerj.12641/supp-8 Supplemental Information 8 Raw data of relative expression levels of 23 pheromone biosynthesis and degradation related genes in *A. yunnanensis*

10.7717/peerj.12641/supp-9 Supplemental Information 9 Nucleotide sequences of the pheromone biosynthesis and degradation related genes in *A. yunnanensis*

10.7717/peerj.12641/supp-10 Supplemental Information 10 Amino acid sequences of the pheromone biosynthesis and degradation related genes in *A. yunnanensis*

10.7717/peerj.12641/supp-11 Supplemental Information 11 FPKM values of the pheromone biosynthesis and degradation related genes in various tissues of *A. yunnanensis*

## Additional Information and Declarations

### Data Availability

The following information was supplied regarding data availability: All raw data of the PG tissues in *A. yunnanensis* are available at NCBI: PRJNA764326. The raw measurements are available in the Supplementary Files.
