# Supplementary figures and images for: Transcriptome analysis identifies candidate genes in the biosynthetic pathway of sex pheromones from a zygaenid moth, Achelura yunnanensis (Lepidoptera: Zygaenidae)
Source: PeerJ. 2021 Dec 14;9:e12641. doi: 10.7717/peerj.12641 (PMC8679906; doi:10.7717/peerj.12641)

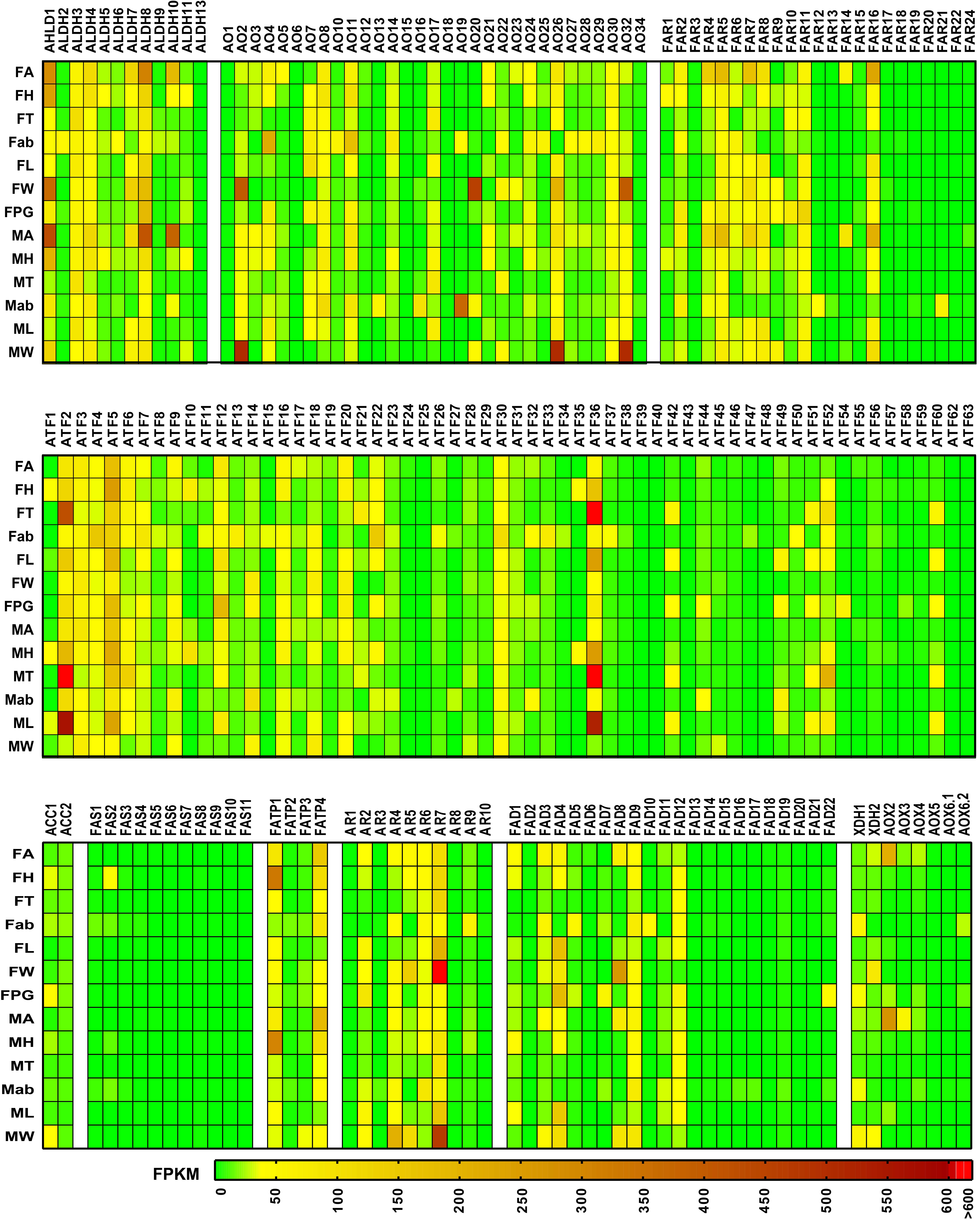

Supplement: Supplemental Information 1 — Different colors denote the expression abundance of genes in tissues as measured by FPKM values. [file peerj-09-12641-s001.jpg]
