# Supplementary material for: Transcriptome analysis identifies candidate genes in the biosynthetic pathway of sex pheromones from a zygaenid moth, Achelura yunnanensis (Lepidoptera: Zygaenidae)
Source: PeerJ. 2021 Dec 14;9:e12641. doi: 10.7717/peerj.12641 (PMC8679906; doi:10.7717/peerj.12641)
Supplement: Supplemental Information 2 [file peerj-09-12641-s002.docx]

**Table S1**

| **Gene** | **Primer sequence (5'-3')** | |
| --- | --- | --- |
|  | **Forward primer** | **Reverse primer** |
| **RT-PCR** | | |
| ACC1 | CTGAGGATCAACGCCAGGAT | AGCGTGTTGACGGTGTTGAG |
| ACC2 | CAACTGCAACACATTGCGGT | AATTCGGCTTCAGTCATCGC |
| ATF2 | GGATTCCAGTCCATCGTCAATAG | CTTATAGTTCTTGGCTGCCTCCT |
| ATF3 | AACCCAATGGATAGGCATACACT | ATCAACAAACTTCCCTGCTTCAC |
| ATF4 | GAGACGTGACCCCGCATAAT | ATCTTCGCATGCGGAACTGT |
| ATF5 | GCTGCCCCAATGTCGTCTAT | CCATCAACGAGCCGAACACT |
| ATF7 | CCTGCTTGTTTTCTTGTTGGAC | AGTACCATGCCAAAGTGCCAG |
| ATF9 | GGTCCTGATGTGCGATAATTGTT | ATTCCTTCCACTCTTTCTTCGCT |
| ATF14 | ACATACACCACGCTCTGTCTTCC | GTCTTCAGAGGTCTCGATCATCG |
| ATF16 | ATCTGTCGGTTCTGCCAATGT | CTTCTAGAACAGCCAACGGGTAC |
| ATF18 | ATCTGGCGTTAGCTTCCTCAGT | GGAGCCAGTGTTCGGTAGATGT |
| ATF20 | ATCGTCTGGTTTAGGAGCATACC | ACTTGAACTAGAGCAAGCCCAAC |
| ATF22 | GTATTAGCAGAGGATCGAGGAGC | CCTCTTTGGAGAGCTCCTTGTAG |
| ATF29 | GCACTGGATGTGACCAGAGATC | GCCCATAAGTTTCCCAGTCCT |
| ATF30 | GTGACCTCATGAACATGCAACAT | CGGTCTTCTCAGTGGCAAAAG |
| ATF36 | CCGTTTTAAAAGCCAGCAAAGT | CGGCAACTCAGGCATAAAGTAGT |
| ATF42 | GTACACCGACTGCTTCCTACTGC | AGGAACTTGTTAGGTTTCCCTGG |
| ATF44 | GATGCCGATACCAGACGATAAG | AGCACCCTAAAGGACGTCTTGT |
| ATF49 | ACTACTCCATGGGCCATTACAGT | CGAAGACGAAACTGGATATCATG |
| ATF51 | ACACCGTATTTCGTTGGTCTCAT | ATGTGATGAGCTAGACTCCGCTT |
| ATF54 | CCGTACTCCTAACCATCGTCTTC | CGACCTGAGTGAGGTTGAAGC |
| ATF58 | ACGACCCGAAGCTCATTAAGAC | GATAAGGAACGGAGACCGTGG |
| ALDH1 | CAAGATTGCTCCAGCTCTTGC | ATCTCTTACCGCCGGCTATG |
| ALDH3 | CAACCCAGCCACAAATGAAGT | GTGGGTCCCGTGTATCACATT |
| ALDH4 | CCACCAGGCGTTGTAAATGTT | CCGTCCTTCACATCAGCAAAT |
| ALDH7 | GCAAGCCGTATCTCATGTCG | CATACTCGAGGTCAGCATCAGC |
| ALDH8 | GTTGTGTACTCCGCTGCCTCT | GTACTAACGGACGTTCCCCCT |
| ALDH10 | ACCCTCTGGCAGAGCGAGTAT | CGTCTGTGGGGTTCACTATCG |
| ALDH11 | GCTCTAATGGGCAATGGTGTAGT | ACCTCCACCAAGTATTTGGGC |
| AO4 | AGAAAGCCATCGACGTAGGCT | AACGAGGGTGCCTAATGGACT |
| AO7 | ACGTCGCATTCCTGGACATAG | ACGCGAGAGGTCAAACAGTTC |
| AO8 | GGCAAATAGCGAAGATCAAGG | ACCACAGCCTTTCCTTTGTTG |
| AO11 | ACTACAAGCATGCCGCTGATT | AGCGGTCTCACGTTGACTTTG |
| AO13 | GACAGGGCAAATAGGTGTTCAG | GGATAAATCCGTCTCGAACTCAT |
| AO16 | CAACAAGTATGGCGTTGGTAGAG | CACCTTCTATTACCCAGGCAGTT |
| AO20 | AGCACAAGGCAGTGTTTAACAAG | GGTAACGTTTCCTCCCTCTATTG |
| AO24 | CCGCTCTACGTTCCACAATG | CAAGCCTCCATCAGTTAAGTCG |
| AO26 | GTGCAACTGGAAGCAGCAGTAG | CTTTGATACCAAGGTTTGCAGC |
| AO30 | CTATTGATTCTCCCCCCAAAAC | CACGATGTTGGATGAGCCATAT |
| AR1 | GGTTAAAGACTGCAGCTGGAGAC | CTATTTCGTTGACTGCCGGC |
| AR2 | CTATACCTCATCCACTGGCCG | GTCGTCCATTGCACTCCAAAG |
| AR4 | CTTTGGTGTAAATCACGCGGT | AAGTTAAGCTCCACTTGCAGGAC |
| AR6 | ACCTCTACTTGATACATTGGCCG | GTTGCAGTCGAAGCTGCTGAT |
| AR7 | CATGCTCGAGATCAGGTAGTTCC | GTCGATGGTACGTATTTCTTCGG |
| AOX2 | CGAATGCATCGCCTTAGCTAC | CGTATCCGGCGTCTGTTCTAG |
| AOX3 | GGAGGTATATGATTCGACGCAAT | GCGAACTTCTGTCGGATCTTTAT |
| AOX4 | ATGGCTACCATCCCATACAAGAG | CCAAGTTCACGTCGACGATAGAT |
| AOX5 | ATATCAGATGCTTTGGGCATAGG | ATCTCCTTCAAGGCCACGTCT |
| AOX6.2 | GGAAAGTGGGAAAAAGAGAGGTT | GAACCTGGTGCTCTACACCAAGT |
| FAD1 | CCAAAGGCAGAGAAACGTCAG | GGTGTAGCGGAAGAGACCACTC |
| FAD2 | ACCATTGACGACACCCCAAG | CACAGCAAAGGCATCAGCAC |
| FAD4 | ATAACTGGAGGAGCTCACCGTC | GCTTTGTAGTCCCAGGGAAATG |
| FAD5 | CCATTCCACAACAGGCTGTTC | CCGATGCTATCCTCTTCACCTC |
| FAD7 | CTGACTGTCGCCTCTCACAACT | CCGGAGTTGTGTCTCTCACTTG |
| FAD8 | CCGTTCATACAGTTCATGCCAC | GTAGTGGTGCGGAGTTTCGTG |
| FAD9 | ATCTGCAAGTTTTGTGAGTCCG | CGTAAGTGTCGTGGAAGTGGAG |
| FAD12 | CTTCTTCTTCTCGCACATCGG | CTTCCTTCGGCAGGTTGTTATC |
| FAR2 | CAGGCGACGAAAGAAACAATAC | CGAATGGGTTCGAAGCAGTAG |
| FAR4 | GGACGGAGTGACACCAAAGATT | GTAACCGTCTACCAACGCTGC |
| FAR5 | CATGAAGTGGCTAAGTGCAAGG | AGATGAACCTCTGCAGCCTGTC |
| FAR7 | GGAGCAGTTGGAGGAAAAGATT | AACGATGTGTGTCAGGAACAGG |
| FAR8 | CATAGCCATGTTCAGACCTGCT | GTTGAAGAGCTTCACGCAGTTG |
| FAR9 | AGCTCTTCAAACCAATGTCCG | CACGTTATCCGATTTTCCACTG |
| FAR10 | GAACCAATTCCAGGCTGGATAG | GTTCTCTTGGTCGCTGATCCTC |
| FAR11 | CTTCTGGGTCTCCATCCAAACT | ACCTGCAGACCTTGGCTAATTC |
| FAR15 | GTTGGGTGGACAATTGGAATG | GTACGACTCGATGTATGCGTCC |
| FAR16 | GCATATTATAAGCGGAGCATGG | GAATCTTCAACGCGTAGTCGAG |
| FAR19 | CTGGAGCCTGTACATGGTTGG | CGAAGGGGAACAGCTGACTATC |
| FAS2 | CGGAGCACTGCGGATCTACT | CGTAGTATGCCGTGGCGTAC |
| FATP1 | CCTTCTTCCTGGAGACCCAAC | CACCCCAATAGTTGGAAGCAG |
| FATP2 | CACTGAACCATTGCCTTGGAC | GCTGGGATTATCCGTGATACG |
| FATP3 | GTCATCTACGGCAACGGAATG | ACCCTCCGTATTCGGTACCAG |
| FATP4 | CAAAACTCTACCAAGGGACGC | CAGGGTGTCCTTGTATCCGAG |
| RPL8 | CCTTGGCAGTAGTTCACTTTCGT | ACCACGAATTCTTCCTGTCCTG |
| **qPCR** | | |
| ATF22 | GGAACACGGAACAAAGAC | TGCTCAAACCCTCAGTAG |
| ATF42 | ACTTGGTCGGTATAACTCTG | TGTATCTGTACTGAGTGGAC |
| ATF54 | GCTTCGTGGTCAGTTATATC | GAGCCATCGCTATTAGAATC |
| ATF58 | TACCTTCTTCTGCCTTGG | CAATGAGACCGATGAACTG |
| ALDH10 | GAAGGTATCAAGGCATTAGC | TCTGGTGAGTCTCTAGTTTG |
| AR2 | CAGAACGAGAAAGAAGTAGG | CAGTGGATGAGGTATAGGTC |
| AR7 | GCTTTAGGCACTTATGGATC | ACATCTCTTATTCCCTCTCC |
| AOX2 | CAACGGAAGGAATAGCAATG | TTGAGTCTCTCGTCGTAATC |
| AOX3 | GTTCTTACTGACCTTCCATC | CTACTCTCATGTTTGCCTTG |
| AOX4 | GTGGATTGAAGAAGAGGAAG | GCATCATCAACACTCAGTAC |
| AOX5 | ATAGATAGTCCAGCGAGAAG | CATCCAATCCATCCTCATTG |
| AOX6.1 | AGTGCAAGTATGCGCGTATTTAC | ATCTTGTGGTATATCCCTGGCC |
| AOX6.2 | TTCTAACGGATAGGACTTGG | CAGACATATTGGAGGTTCAC |
| FAD4 | CATACGAAAGTGGGAGAAAC | CAGCCATATAACCCATCAAC |
| FAD5 | GTCGTCCTAAAGTTGTTTGG | CATTGAAGGTGATCCAGTTG |
| FAD7 | TCCTACTTCCTCTGTTGATG | TATCTCTGGATGGTGATGAG |
| FAD12 | CCTCTATGCTGACCCTATAC | CATTCGTCCATGTCTCATTC |
| FAR1 | ATACGTTGATACGAGGGAAG | CCACTATGGGCACTATTTTG |
| FAR9 | GTATAGGACGAAAGCCAAAG | GAAGTCTTGTTCCAGAGTTC |
| FAR10 | GAACCTAAGTTGATCGGAAG | CCAATGCTGCGATTACTATC |
| FAR11 | GGTCTTTCTCTTGTCCAAAC | GGCACCATATTCCTATCTTC |
| FAR12 | GTGTTATCACTGGACATGAC | CTGGATCTGGTTGAATGATG |
| FAR19 | GTACATGGTTGGGTTGATAG | CTGCTACTATGCTGAGATTC |
| FAR20 | AGGTGAAGACATCGTAAGAG | ACCTCCACCAACTATAATCC |
| RPS4 | GATTGTGTCCCGTGAAAG | AGTGAGATGTACGCCTTC |
