# Supplementary material for: Transcriptome analysis identifies candidate genes in the biosynthetic pathway of sex pheromones from a zygaenid moth, Achelura yunnanensis (Lepidoptera: Zygaenidae)
Source: PeerJ. 2021 Dec 14;9:e12641. doi: 10.7717/peerj.12641 (PMC8679906; doi:10.7717/peerj.12641)
Supplement: Supplemental Information 5 [file peerj-09-12641-s005.docx]

**Table S4**

| **Gene** | **ORF (AA)** | **Full length** | **NCBI blast hit (References/Name/Species)** | **E value** | **Identity (%)** |
| --- | --- | --- | --- | --- | --- |
| **Acetyl-CoA carboxylase (ACC)** | | | | | |
| ACC1 | 7068 | Yes | QLI61955.1 acetyl-CoA carboxylase 2 [*Streltzoviella insularis*] | 0.0 | 88.17 |
| ACC2 | 2115 | Yes | XP_023945828.1 methylcrotonoyl-CoA carboxylase subunit alpha, mitochondrial [*Bicyclus anynana*] | 0.0 | 76.84 |
| ACC3 | 429 | No | AOD74995.1 acetyl-CoA carboxylase 2 [*Helicoverpa armigera*] | 8e-36 | 68.81 |
| **Fatty acid synthase (FAS)** | | | | | |
| FAS1 | 6897 | Yes | XP_013167810.1 PREDICTED: fatty acid synthase [*Papilio xuthus*] | 0.0 | 44.32 |
| FAS2 | 7263 | Yes | XP_026734139.1 fatty acid synthase isoform X1 [*Trichoplusia ni*] | 0.0 | 74.16 |
| FAS3 | 7266 | Yes | XP_030021952.1 fatty acid synthase [*Manduca sexta*] | 0.0 | 80.46 |
| FAS4 | 7038 | Yes | XP_028028986.1 fatty acid synthase-like [*Bombyx mandarina*] | 0.0 | 56.80 |
| FAS5 | 1713 | No | KAF9424205.1 hypothetical protein HW555_000598 [*Spodoptera* *exigua*] | 2e-166 | 45.25 |
| FAS6 | 1287 | No | XP_035437791.1 fatty acid synthase-like [*Spodoptera frugiperda*] | 1e-127 | 47.95 |
| FAS7 | 1311 | No | XP_021181026.1 fatty acid synthase-like [*Helicoverpa armigera*] | 0.0 | 70.64 |
| FAS8 | 6201 | No | AKD01761.1 fatty acid synthase 2, partial [*Helicoverpa assulta*] | 0.0 | 70.62 |
| FAS9 | 1323 | No | PCG75470.1 hypothetical protein B5V51_11633 [*Heliothis virescens*] | 3e-149 | 48.84 |
| FAS10 | 615 | No | XP_013196463.1 PREDICTED: fatty acid synthase-like [*Amyelois transitella*] | 6e-63 | 49.76 |
| FAS11 | 1155 | No | RVE49259.1 hypothetical protein evm_006053 [*Chilo suppressalis*] | 2e-153 | 61.29 |
| FAS12 | 513 | No | KPI99075.1 Fatty acid synthase [*Papilio xuthus*] | 1e-85 | 73.68 |
| **Acetyltransferase (ATF)** | | | | | |
| ATF1 | 1227 | Yes | XP_037295331.1 nose resistant to fluoxetine protein 6 [*Manduca sexta*] | 6e-95 | 40.54 |
| ATF2 | 1191 | Yes | AGG54999.1 thiolase 1 [*Heliothis virescens*] | 0.0 | 82.07 |
| ATF3 | 1611 | Yes | XP_032518792.1 non-specific lipid-transfer protein [*Danaus plexippus plexippus*] | 0.0 | 87.13 |
| ATF4 | 532 | Yes | XP_026332522.1 probable N-acetyltransferase san [*Hyposmocoma kahamanoa*] | 4e-122 | 94.32 |
| ATF5 | 1404 | Yes | XP_030034270.2 dihydrolipoyllysine-residue succinyltransferase component of 2-oxoglutarate dehydrogenase complex, mitochondrial [*Manduca sexta*] | 0.0 | 84.83 |
| ATF6 | 1500 | Yes | XP_038222365.1dihydrolipoyllysine-residue acetyltransferase component of pyruvate dehydrogenase complex, mitochondrial isoform X1 [*Zerene cesonia*] | 0.0 | 75.20 |
| ATF7 | 1431 | Yes | XP_035436906.1 lysophospholipid acyltransferase 5-like [*Spodoptera frugiperda*] | 0.0 | 65.90 |
| ATF8 | 1956 | Yes | XP_026752133.1 carnitine O-palmitoyltransferase 2, mitochondrial [*Galleria mellonella*] | 0.0 | 73.35 |
| ATF9 | 1443 | Yes | XP_035454014.1 glycerol-3-phosphate acyltransferase 4-like isoform X2 [*Spodoptera* *frugiperda*] | 0.0 | 83.68 |
| ATF10 | 855 | Yes | XP_028176584.1 1-acyl-sn-glycerol-3-phosphate acyltransferase alpha [*Ostrinia furnacalis*] | 2e-179 | 85.66 |
| ATF11 | 2613 | Yes | XP_026322853.1 glycerol-3-phosphate acyltransferase 1, mitochondrial isoform X1 [*Hyposmocoma kahamanoa*] | 0.0 | 64.13 |
| ATF12 | 1842 | Yes | XP_013161772.1 PREDICTED: nose resistant to fluoxetine protein 6 isoform X1 [*Papilio* *xuthus*] | 0.0 | 75.17 |
| ATF13 | 885 | Yes | PCG78901.1 hypothetical protein B5V51_2782 [*Heliothis virescens*] | 7e-139 | 70.20 |
| ATF14 | 822 | Yes | XP_023939062.11-acyl-sn-glycerol-3-phosphate acyltransferase alpha-like [*Bicyclus anynana*] | 1e-143 | 71.85 |
| ATF15 | 1065 | Yes | XP_030022877.1 AB hydrolase superfamily protein YfhM-like [*Manduca sexta*] | 7e-161 | 63.03 |
| ATF16 | 1515 | Yes | XP_026750436.1 lysophospholipid acyltransferase 2 [*Galleria* *mellonella*] | 0.0 | 77.51 |
| ATF17 | 1062 | Yes | XP_038222891.1 2-acylglycerol O-acyltransferase 2-like [*Zerene cesonia*] | 3e-174 | 67.15 |
| ATF18 | 1407 | Yes | XP_013182360.1 PREDICTED: sterol O-acyltransferase 2 [*Papilio xuthus*] | 0.0 | 72.51 |
| ATF19 | 1131 | Yes | XP_035434954.1 1-acylglycerol-3-phosphate O-acyltransferase ABHD5-like isoform X1 [*Spodoptera frugiperda*] | 0.0 | 83.42 |
| ATF20 | 1458 | Yes | XP_021192342.1 lysophospholipid acyltransferase 7-like [*Helicoverpa armigera*] | 0.0 | 80.00 |
| ATF21 | 1239 | Yes | PCG80023.1 hypothetical protein B5V51_12259 [*Heliothis virescens*] | 0.0 | 82.52 |
| ATF22 | 849 | Yes | XP_022835069.1 1-acyl-sn-glycerol-3-phosphate acyltransferase alpha-like [*Spodoptera litura*] | 1e-173 | 83.93 |
| ATF23 | 1152 | Yes | ARD71217.1 acetyltransferase [*Spodoptera exigua*] | 0.0 | 80.94 |
| ATF24 | 1953 | Yes | XP_022826357.1 choline O-acetyltransferase [*Spodoptera litura*] | 0.0 | 85.32 |
| ATF25 | 1938 | Yes | XP_030034481.1 O-acyltransferase like protein isoform X1 [*Manduca sexta*] | 0.0 | 41.86 |
| ATF26 | 1476 | Yes | XP_013188841.1PREDICTED: diacylglycerol O-acyltransferase 1 isoform X1 [*Amyelois transitella*] | 0.0 | 79.35 |
| ATF27 | 1425 | Yes | ARD71207.1 acetyltransferase [*Spodoptera exigua*] | 0.0 | 70.35 |
| ATF28 | 1896 | Yes | XP_026329564.1 carnitine O-acetyltransferase-like isoform X2 [*Hyposmocoma kahamanoa*] | 0.0 | 71.56 |
| ATF29 | 729 | Yes | XP_028179577.1 uncharacterized protein LOC114366796 [*Ostrinia furnacalis*] | 1e-109 | 64.58 |
| ATF30 | 534 | Yes | XP_038223576.1 N-alpha-acetyltransferase 10 [*Zerene* *cesonia*] | 6e-124 | 94.41 |
| ATF31 | 2319 | Yes | XP_028162157.1 carnitine O-palmitoyltransferase 1, liver isoform [*Ostrinia* *furnacalis*] | 0.0 | 85.32 |
| ATF32 | 696 | Yes | KAF9412738.1 hypothetical protein HW555_008823 [*Spodoptera* *exigua*] | 5e-83 | 54.31 |
| ATF33 | 2076 | Yes | AIN34685.1 fatty alcohol acetyltransferase [*Agrotis* *segetum*] | 0.0 | 91.65 |
| ATF34 | 2007 | Yes | PCG75641.1 hypothetical protein B5V51_11260 [*Heliothis* *virescens*] | 0.0 | 61.35 |
| ATF35 | 1287 | Yes | XP_022827940.1 lysocardiolipin acyltransferase 1-like [*Spodoptera* *litura*] | 0.0 | 66.75 |
| ATF36 | 1422 | Yes | XP_026744850.1 trifunctional enzyme subunit beta, mitochondrial-like [*Trichoplusia* *ni*] | 0.0 | 87.76 |
| ATF37 | 1164 | Yes | KAF9799770.1 hypothetical protein SFRURICE_005063 [*Spodoptera* *frugiperda*] | 2e-157 | 54.77 |
| ATF38 | 744 | Yes | XP_034831515.1 N-alpha-acetyltransferase 60 [*Maniola* *hyperantus*] | 1e-165 | 91.09 |
| ATF39 | 960 | Yes | XP_035434170.1 nose resistant to fluoxetine protein 6-like [*Spodoptera* *frugiperda*] | 3e-71 | 39.00 |
| ATF40 | 699 | Yes | KAF9412738.1 hypothetical protein HW555_008823 [*Spodoptera* *exigua*] | 8e-79 | 51.52 |
| ATF41 | 2208 | Yes | KAF9423095.1 hypothetical protein HW555_001399 [*Spodoptera* *exigua*] | 0.0 | 44.86 |
| ATF42 | 2085 | Yes | XP_035431218.1 uncharacterized protein LOC118263383 [*Spodoptera* *frugiperda*] | 0.0 | 59.14 |
| ATF43 | 1911 | Yes | XP_012548289.2 O-acyltransferase like protein isoform X5 [*Bombyx* *mori*] | 0.0 | 63.35 |
| ATF44 | 951 | Yes | XP_030035622.1 glycerol-3-phosphate acyltransferase 3 isoform X1 [*Manduca* *sexta*] | 2e-162 | 75.42 |
| ATF45 | 864 | Yes | XP_028178708.1 lysosomal thioesterase PPT2 homolog [*Ostrinia* *furnacalis*] | 3e-174 | 82.92 |
| ATF46 | 1131 | Yes | XP_037293301.1 acyl-CoA:lysophosphatidylglycerol acyltransferase 1-like isoform X2 [*Manduca* *sexta*] | 0.0 | 77.98 |
| ATF47 | 1899 | Yes | XP_037295331.1 nose resistant to fluoxetine protein 6 [*Manduca* *sexta*] | 9e-167 | 40.92 |
| ATF48 | 1773 | Yes | XP_037295331.1 nose resistant to fluoxetine protein 6 [*Manduca* *sexta*] | 6e-158 | 40.50 |
| ATF49 | 1836 | Yes | XP_026313458.1 O-acyltransferase like protein-like isoform X1 [*Hyposmocoma* *kahamanoa*] | 0.0 | 46.21 |
| ATF50 | 549 | Yes | XP_030020229.1 dynactin subunit 5 [*Manduca* *sexta*] | 2e-129 | 96.70 |
| ATF51 | 2343 | Yes | XP_022828881.1 nose resistant to fluoxetine protein 6-like [*Spodoptera* *litura*] | 0.0 | 57.68 |
| ATF52 | 1548 | Yes | XP_031766647.1 acetyl-CoA acetyltransferase, mitochondrial isoform X1 [*Galleria* *mellonella*] | 0.0 | 87.06 |
| ATF53 | 1080 | Yes | XP_028034782.1 dihydrolipoyllysine-residue succinyltransferase component of 2-oxoglutarate dehydrogenase complex, mitochondrial-like [*Bombyx* *mandarina*] | 0.0 | 83.98 |
| ATF54 | 1863 | Yes | XP_011565397.2 O-acyltransferase like protein-like [*Plutella* *xylostella*] | 0.0 | 47.12 |
| ATF55 | 1917 | Yes | XP_013137589.1 PREDICTED: nose resistant to fluoxetine protein 6-like [*Papilio* *polytes*] | 0.0 | 59.74 |
| ATF56 | 2007 | Yes | XP_026738485.1 cysteine-rich protein 2-binding protein [*Trichoplusia* *ni*] | 0.0 | 71.03 |
| ATF57 | 708 | Yes | XP_022125365.1 uncharacterized protein LOC111000285 [*Pieris* *rapae*] | 1e-123 | 72.22 |
| ATF58 | 2610 | Yes | XP_022113117.1 nose resistant to fluoxetine protein 6-like [*Pieris* *rapae*] | 0.0 | 74.57 |
| ATF59 | 2319 | Yes | XP_026484393.1 nose resistant to fluoxetine protein 6-like [*Vanessa* *tameamea*] | 0.0 | 63.12 |
| ATF60 | 3678 | No | XP_035431218.1 uncharacterized protein LOC118263383 [*Spodoptera* *frugiperda*] | 0.0 | 62.97 |
| ATF61 | 1087 | No | XP_021186304.1 nose resistant to fluoxetine protein 6-like [*Helicoverpa* *armigera*] | 5e-84 | 45.25 |
| ATF62 | 1845 | No | XP_028161295.1 O-acyltransferase like protein-like isoform X1 [*Ostrinia* *furnacalis*] | 0.0 | 48.12 |
| ATF63 | 1404 | No | XP_030034482.1 O-acyltransferase like protein isoform X2 [*Manduca* *sexta*] | 1e-144 | 45.70 |
| **Aldehyde dehydrogenase (ALDH)** | | | | | |
| ALDH1 | 1320 | Yes | XP_021182274.1 aldehyde dehydrogenase X, mitochondrial-like [*Helicoverpa* *armigera*] | 0.0 | 83.37 |
| ALDH2 | 1641 | Yes | XP_023939734.1 uncharacterized protein LOC112047054 [*Bicyclus* *anynana*] | 6e-65 | 30.30 |
| ALDH3 | 1569 | Yes | XP_023952753.1 probable methylmalonate-semialdehyde dehydrogenase [acylating], mitochondrial [*Bicyclus* *anynana*] | 0.0 | 89.46 |
| ALDH4 | 1467 | Yes | XP_026315283.1 aldehyde dehydrogenase X, mitochondrial-like [*Hyposmocoma* *kahamanoa*] | 0.0 | 85.45 |
| ALDH5 | 1524 | Yes | XP_028162948.1 succinate-semialdehyde dehydrogenase, mitochondrial [*Ostrinia* *furnacalis*] | 0.0 | 74.95 |
| ALDH6 | 1611 | Yes | XP_004930408.1 putative aldehyde dehydrogenase family 7 member A1 homolog [*Bombyx* *mori*] | 0.0 | 85.42 |
| ALDH7 | 1557 | Yes | XP_021201072.1 aldehyde dehydrogenase, mitochondrial [*Helicoverpa* *armigera*] | 0.0 | 76.81 |
| ALDH8 | 1719 | Yes | XP_034835423.1 aldehyde dehydrogenase, dimeric NADP-preferring isoform X2 [*Maniola* *hyperantus*] | 0.0 | 70.67 |
| ALDH9 | 1620 | Yes | XP_039759278.1 uncharacterized protein LOC120633201 isoform X1 [*Pararge* *aegeria*] | 9e-66 | 30.51 |
| ALDH10 | 2784 | Yes | XP_022831585.1 cytosolic 10-formyltetrahydrofolate dehydrogenase [*Spodoptera* *litura*] | 0.0 | 86.08 |
| ALDH11 | 1701 | Yes | XP_030035609.2 delta-1-pyrroline-5-carboxylate dehydrogenase, mitochondrial [*Manduca* *sexta*] | 0.0 | 79.86 |
| ALDH12 | 366 | No | XP_037296330.1 glutarate-semialdehyde dehydrogenase [*Manduca* *sexta*] | 8e-45 | 61.16 |
| ALDH13 | 801 | No | XP_028162948.1 succinate-semialdehyde dehydrogenase, mitochondrial [*Ostrinia* *furnacalis*] | 3e-145 | 78.28 |
| **Alcohol oxidase/dehydrogenase (AO/ADH)** | | | | | |
| AO1 | 1038 | Yes | XP_038222127.1 sorbitol dehydrogenase-like [*Zerene* *cesonia*] | 1e-178 | 67.83 |
| AO2 | 792 | Yes | QLI62149.1 alcohol dehydrogenase 8 [*Streltzoviella* *insularis*] | 8e-85 | 50.95 |
| AO3 | 1179 | Yes | XP_037294197.1 1,5-anhydro-D-fructose reductase-like [*Manduca* *sexta*] | 0.0 | 75.15 |
| AO4 | 945 | Yes | QLI62145.1 alcohol dehydrogenase 4 [*Streltzoviella* *insularis*] | 2e-149 | 64.33 |
| AO5 | 1008 | Yes | XP_037293749.1 prostaglandin reductase 1-like [*Manduca* *sexta*] | 2e-176 | 71.43 |
| AO6 | 822 | Yes | AKQ06147.1 alcohol dehydrogenase AD1 [*Cydia* *pomonella*] | 3e-108 | 59.41 |
| AO7 | 660 | Yes | XP_021193750.1 15-hydroxyprostaglandin dehydrogenase [NAD(+)]-like [*Helicoverpa* *armigera*] | 3e-94 | 65.75 |
| AO8 | 1005 | Yes | KPJ08829.1 Prostaglandin reductase 1 [*Papilio* *machaon*] | 0.0 | 74.63 |
| AO9 | 765 | Yes | KOB65394.1 Alcohol dehydrogenase [*Operophtera* *brumata*] | 2e-86 | 50.79 |
| AO10 | 1041 | Yes | XP_012546333.1 zinc-type alcohol dehydrogenase-like protein SERP1785 [*Bombyx* *mori*] | 0.0 | 73.24 |
| AO11 | 1086 | Yes | XP_038222008.1 sorbitol dehydrogenase-like [*Zerene* *cesonia*] | 0.0 | 84.64 |
| AO12 | 1029 | Yes | XP_028178468.1 D-arabinitol dehydrogenase 1-like [*Ostrinia* *furnacalis*] | 0.0 | 76.90 |
| AO13 | 759 | Yes | KOB65394.1 Alcohol dehydrogenase [*Operophtera* *brumata*] | 2e-76 | 50.62 |
| AO14 | 771 | Yes | XP_026761478.1 3-hydroxyacyl-CoA dehydrogenase type-2-like isoform X1 [*Galleria* *mellonella*] | 5e-157 | 81.64 |
| AO15 | 1008 | Yes | XP_037293749.1 prostaglandin reductase 1-like [*Manduca* *sexta*] | 2e-171 | 69.64 |
| AO16 | 786 | Yes | XP_023943578.1 15-hydroxyprostaglandin dehydrogenase [NAD(+)]-like [*Bicyclus* *anynana*] | 2e-90 | 53.44 |
| AO17 | 1074 | Yes | AKQ06152.1 alcohol dehydrogenase AD6 [*Cydia* *pomonella*] | 0.0 | 74.93 |
| AO18 | 993 | Yes | QLI62147.1 alcohol dehydrogenase 6 [*Streltzoviella* *insularis*] | 2e-143 | 58.79 |
| AO19 | 768 | Yes | AKQ06148.1 alcohol dehydrogenase AD2 [*Cydia* *pomonella*] | 8e-100 | 58.17 |
| AO20 | 789 | Yes | XP_026753012.1 15-hydroxyprostaglandin dehydrogenase [NAD(+)]-like [*Galleria* *mellonella*] | 2e-78 | 48.80 |
| AO21 | 762 | Yes | XP_034830405.1 15-hydroxyprostaglandin dehydrogenase [NAD(+)]-like isoform X2 [*Maniola* *hyperantus*] | 3e-94 | 52.19 |
| AO22 | 1440 | Yes | QLI62146.1 alcohol dehydrogenase 5 [*Streltzoviella* *insularis*] | 0.0 | 64.34 |
| AO23 | 786 | Yes | QLI62149.1 alcohol dehydrogenase 8 [*Streltzoviella* *insularis*] | 2e-99 | 55.98 |
| AO24 | 1131 | Yes | XP_022829789.1 alcohol dehydrogenase class-3 [*Spodoptera* *litura*] | 0.0 | 94.15 |
| AO25 | 759 | Yes | XP_013146841.1 PREDICTED: 15-hydroxyprostaglandin dehydrogenase [NAD(+)]-like [*Papilio* *polytes*] | 8e-165 | 85.71 |
| AO26 | 978 | Yes | AKD01753.1 alcohol dehydrogenase 15, partial [*Helicoverpa* *assulta*] | 3e-177 | 73.15 |
| AO27 | 1017 | Yes | PCG73369.1 hypothetical protein B5V51_14885 [*Heliothis* *virescens*] | 0.0 | 75.75 |
| AO28 | 1032 | Yes | XP_012546333.1 zinc-type alcohol dehydrogenase-like protein SERP1785 [*Bombyx* *mori*] | 1e-180 | 70.59 |
| AO29 | 1104 | Yes | XP_028166785.1 enoyl-[acyl-carrier-protein] reductase, mitochondrial [*Ostrinia* *furnacalis*] | 0.0 | 75.35 |
| AO30 | 1365 | Yes | XP_037873372.1 vesicle amine transport protein isoform X2 [*Bombyx* *mori*] | 0.0 | 91.20 |
| AO31 | 768 | Yes | KOB65394.1 Alcohol dehydrogenase [*Operophtera* *brumata*] | 4e-76 | 51.81 |
| AO32 | 819 | Yes | AKQ06147.1 alcohol dehydrogenase AD1 [*Cydia* *pomonella*] | 2e-112 | 60.52 |
| AO33 | 339 | No | XP_026485267.1 15-hydroxyprostaglandin dehydrogenase [NAD(+)]-like [*Vanessa* *tameamea*] | 5e-76 | 96.46 |
| AO34 | 258 | No | XP_013185472.1 PREDICTED: prostaglandin reductase 1-like [*Amyelois* *transitella*] | 7e-34 | 67.06 |
| **Aldehyde reductase (AR)** | | | | | |
| AR1 | 630 | Yes | XP_026333799.1 aldo-keto reductase AKR2E4-like [*Hyposmocoma* *kahamanoa*] | 2e-90 | 70.92 |
| AR2 | 912 | Yes | XP_039762118.1 aldo-keto reductase family 1 member B1-like [*Pararge* *aegeria*] | 5e-176 | 77.23 |
| AR3 | 939 | Yes | XP_011562926.2 aldo-keto reductase AKR2E4-like [*Plutella* *xylostella*] | 1e-135 | 58.90 |
| AR4 | 999 | Yes | XP_035455346.1 aldo-keto reductase AKR2E4-like isoform X1 [*Spodoptera* *frugiperda*] | 4e-167 | 66.87 |
| AR5 | 1005 | Yes | XP_021198110.1 aldo-keto reductase AKR2E4-like [*Helicoverpa* *armigera*] | 1e-152 | 66.67 |
| AR6 | 957 | Yes | XP_038221005.1 aldo-keto reductase family 1 member B1-like isoform X2 [*Zerene* *cesonia*] | 0.0 | 80.19 |
| AR7 | 930 | Yes | XP_026333799.1 aldo-keto reductase AKR2E4-like [*Hyposmocoma* *kahamanoa*] | 9e-147 | 66.99 |
| AR8 | 1032 | Yes | XP_030031946.2 aldo-keto reductase AKR2E4 isoform X2 [*Manduca* *sexta*] | 4e-165 | 64.14 |
| AR9 | 918 | Yes | XP_028177527.1 aldo-keto reductase AKR2E4-like [*Ostrinia* *furnacalis*] | 1e-167 | 74.10 |
| AR10 | 3828 | Yes | XP_021199334.1 aldose reductase-like isoform X1 [*Helicoverpa* *armigera*] | 0.0 | 63.02 |
| **Fatty acyl desaturase (FAD)** | | | | | |
| FAD1 | 1047 | Yes | QLI61972.1 desaturase 17 [*Streltzoviella* *insularis*] | 0.0 | 80.74 |
| FAD2 | 1113 | Yes | XP_037975231.1 stearoyl-CoA desaturase 5-like [*Plutella* *xylostella*] | 0.0 | 77.63 |
| FAD3 | 1014 | Yes | ARD71185.1 desaturase [*Spodoptera* *exigua*] | 2e-175 | 68.48 |
| FAD4 | 1095 | Yes | XP_013193663.1 PREDICTED: acyl-CoA Delta(11) desaturase [*Amyelois* *transitella*] | 4e-153 | 58.36 |
| FAD5 | 996 | Yes | QMX41398.1 desaturase DES7 [*Dioryctria* *abietella*] | 0.0 | 92.83 |
| FAD6 | 1326 | Yes | XP_026321814.1 cytochrome b5-related protein-like isoform X1 [*Hyposmocoma* *kahamanoa*] | 0.0 | 61.61 |
| FAD7 | 1359 | Yes | XP_004924008.1 cytochrome b5-related protein [*Bombyx* *mori*] | 0.0 | 71.68 |
| FAD8 | 1338 | Yes | XP_026496803.1 cytochrome b5-related protein-like [*Vanessa* *tameamea*] | 0.0 | 62.20 |
| FAD9 | 1011 | Yes | PCG79838.1 hypothetical protein B5V51_13516 [*Heliothis* *virescens*] | 0.0 | 77.88 |
| FAD10 | 1107 | Yes | XP_035433488.1 acyl-CoA Delta(11) desaturase-like [*Spodoptera* *frugiperda*] | 7e-163 | 60.43 |
| FAD11 | 1095 | Yes | QLI61964.1 desaturase 9 [*Streltzoviella* *insularis*] | 0.0 | 71.66 |
| FAD12 | 1059 | Yes | XP_032515470.1 acyl-CoA Delta(11) desaturase-like isoform X1 [*Danaus* *plexippus* *plexippus*] | 0.0 | 85.51 |
| FAD13 | 1197 | Yes | XP_026757907.1 stearoyl-CoA desaturase 5-like [*Galleria* *mellonella*] | 0.0 | 70.38 |
| FAD14 | 1092 | Yes | XP_035440106.1 acyl-CoA Delta(11) desaturase-like [*Spodoptera* *frugiperda*] | 0.0 | 85.63 |
| FAD15 | 921 | No | AGO45839.1 acyl-CoA desaturase HvirLPAQ [*Heliothis* *virescens*] | 5e-159 | 66.56 |
| FAD16 | 600 | No | QLI61971.1 desaturase 16 [*Streltzoviella* *insularis*] | 9e-105 | 70.00 |
| FAD17 | 498 | No | KOB69615.1 Terminal desaturase [*Operophtera* *brumata*] | 5e-79 | 68.59 |
| FAD18 | 816 | No | XP_026741305.1 acyl-CoA Delta(11) desaturase-like [*Trichoplusia* **ni**] | 2e-161 | 76.38 |
| FAD19 | 1230 | No | XP_026321814.1 cytochrome b5-related protein-like isoform X1 [*Hyposmocoma* *kahamanoa*] | 3e-177 | 57.71 |
| FAD20 | 756 | No | XP_026496803.1 cytochrome b5-related protein-like [*Vanessa* *tameamea*] | 4e-107 | 57.77 |
| FAD21 | 861 | No | XP_028034399.1 acyl-CoA Delta(11) desaturase-like isoform X1 [*Bombyx* *mandarina*] | 2e-163 | 72.79 |
| FAD22 | 531 | No | XP_011550465.2 cytochrome b5-related protein-like [*Plutella* *xylostella*] | 8e-84 | 68.36 |
| **Fatty acyl-CoA reductase (FAR)** | | | | | |
| FAR1 | 1353 | Yes | AFD04728.1 fatty acid reductase [*Helicoverpa armigera*] | 3e-126 | 42.83 |
| FAR2 | 1551 | Yes | KAF9421846.1 hypothetical protein HW555_002286 [*Spodoptera exigua*] | 0.0 | 78.29 |
| FAR3 | 1476 | Yes | XP_037977368.1 putative fatty acyl-CoA reductase CG5065 isoform X1 [*Plutella xylostella*] | 0.0 | 52.26 |
| FAR4 | 1872 | Yes | ADI82775.1 fatty-acyl CoA reductase 2 [*Ostrinia nubilalis*] | 0.0 | 66.51 |
| FAR5 | 1533 | Yes | XP_035456003.1 putative fatty acyl-CoA reductase CG8306 [*Spodoptera frugiperda*] | 0.0 | 74.12 |
| FAR6 | 1584 | Yes | XP_030041049.2 putative fatty acyl-CoA reductase CG5065 [*Manduca sexta*] | 0.0 | 79.77 |
| FAR7 | 1500 | Yes | AKD01774.1 fatty acyl-CoA reductase 13, partial [*Helicoverpa armigera*] | 0.0 | 61.17 |
| FAR8 | 1596 | Yes | XP_014371683.1 PREDICTED: fatty acyl-CoA reductase 2-like [*Papilio machaon*] | 0.0 | 69.89 |
| FAR9 | 1719 | Yes | PCG69973.1 hypothetical protein B5V51_3492 [*Heliothis* *virescens*] | 0.0 | 71.07 |
| FAR10 | 1566 | Yes | XP_013143320.1 PREDICTED: fatty acyl-CoA reductase 1-like [*Papilio polytes*] | 0.0 | 66.27 |
| FAR11 | 1554 | Yes | XP_039763614.1 putative fatty acyl-CoA reductase CG5065 [*Pararge aegeria*] | 0.0 | 72.67 |
| FAR12 | 1377 | Yes | XP_032515546.1 putative fatty acyl-CoA reductase CG5065 [*Danaus plexippus plexippus*] | 2e-171 | 54.55 |
| FAR13 | 1608 | Yes | XP_013143317.1 PREDICTED: fatty acyl-CoA reductase 1-like [*Papilio polytes*] | 0.0 | 54.53 |
| FAR14 | 1560 | Yes | XP_034827906.1 putative fatty acyl-CoA reductase CG5065 [*Maniola hyperantus*] | 0.0 | 81.53 |
| FAR15 | 1572 | Yes | XP_026483533.1 putative fatty acyl-CoA reductase CG5065 [*Vanessa tameamea*] | 0.0 | 89.60 |
| FAR16 | 1551 | Yes | XP_037977374.1 putative fatty acyl-CoA reductase CG5065 isoform X1 [*Plutella xylostella*] | 0.0 | 64.41 |
| FAR17 | 1572 | Yes | XP_013176193.1 PREDICTED: fatty acyl-CoA reductase 1-like [*Papilio xuthus*] | 0.0 | 56.50 |
| FAR18 | 1587 | Yes | XP_026483533.1 putative fatty acyl-CoA reductase CG5065 [*Vanessa tameamea*] | 0.0 | 69.05 |
| FAR19 | 1368 | Yes | XP_032515546.1 putative fatty acyl-CoA reductase CG5065 [*Danaus plexippus plexippus*] | 4e-175 | 53.29 |
| FAR20 | 1152 | No | XP_013143317.1 PREDICTED: fatty acyl-CoA reductase 1-like [*Papilio* *polytes*] | 5e-169 | 61.20 |
| FAR21 | 1491 | No | XP_013176193.1 PREDICTED: fatty acyl-CoA reductase 1-like [*Papilio xuthus*] | 0.0 | 55.67 |
| FAR22 | 1245 | No | XP_014369376.1 PREDICTED: fatty acyl-CoA reductase 1-like [*Papilio machaon*] | 6e-113 | 40.38 |
| FAR23 | 342 | No | XP_026483531.1 fatty acyl-CoA reductase wat-like [*Vanessa tameamea*] | 3e-47 | 65.79 |
| FAR24 | 348 | No | XP_013177221.1 fatty acyl-CoA reductase 8 [*Streltzoviella* *insularis*] | 6e-66 | 88.79 |
| **Fatty acid transport protein (FATP)** | | | | | |
| FATP1 | 1983 | Yes | XP_026750413.1 long-chain fatty acid transport protein 1 [*Galleria mellonella*] | 0.0 | 76.10 |
| FATP2 | 2082 | Yes | PCG80606.1 hypothetical protein B5V51_5556 [*Heliothis virescens*] | 0.0 | 71.45 |
| FATP3 | 1968 | Yes | RVE51224.1 hypothetical protein evm_004189 [*Chilo suppressalis*] | 0.0 | 72.52 |
| FATP4 | 1932 | Yes | XP_013181333.1 PREDICTED: long-chain fatty acid transport protein 4-like [*Papilio xuthus*] | 0.0 | 85.07 |
| **Xanthine dehydrogenase (XDH) and aldehyde oxidase (AOX)** | | | | | |
| XDH1 | 4056 | Yes | XP_030020628.1 xanthine dehydrogenase [*Manduca* *sexta*] | 0.0 | 80.81 |
| XDH2 | 4017 | Yes | XP_021182356.1 xanthine dehydrogenase-like [*Helicoverpa* *armigera*] | 0.0 | 66.47 |
| AOX2 | 3903 | Yes | XP_037293400.1 xanthine dehydrogenase isoform X1 [*Manduca* *sexta*] | 0.0 | 68.62 |
| AOX3 | 3612 | Yes | QLI62139.1 aldehyde oxidase 8 [*Streltzoviella* *insularis*] | 0.0 | 67.68 |
| AOX4 | 3783 | Yes | AKQ06145.1 aldehyde oxidase AOX1 [*Cydia pomonella*] | 0.0 | 63.98 |
| AOX5 | 3720 | Yes | XP_028164012.1 xanthine dehydrogenase-like [*Ostrinia furnacalis*] | 0.0 | 61.68 |
| AOX6.1 | 3795 | Yes | XP_026741742.1 xanthine dehydrogenase/oxidase-like [*Trichoplusia ni*] | 0.0 | 58.58 |
| AOX6.2 | 3771 | Yes | XP_026741881.1 xanthine dehydrogenase-like [*Trichoplusia ni*] | 0.0 | 58.59 |
