# Supplementary material for: Transcriptome analysis identifies candidate genes in the biosynthetic pathway of sex pheromones from a zygaenid moth, Achelura yunnanensis (Lepidoptera: Zygaenidae)
Source: PeerJ. 2021 Dec 14;9:e12641. doi: 10.7717/peerj.12641 (PMC8679906; doi:10.7717/peerj.12641)
Supplement: Supplemental Information 6 [file peerj-09-12641-s006.pptx]

## Slide 1
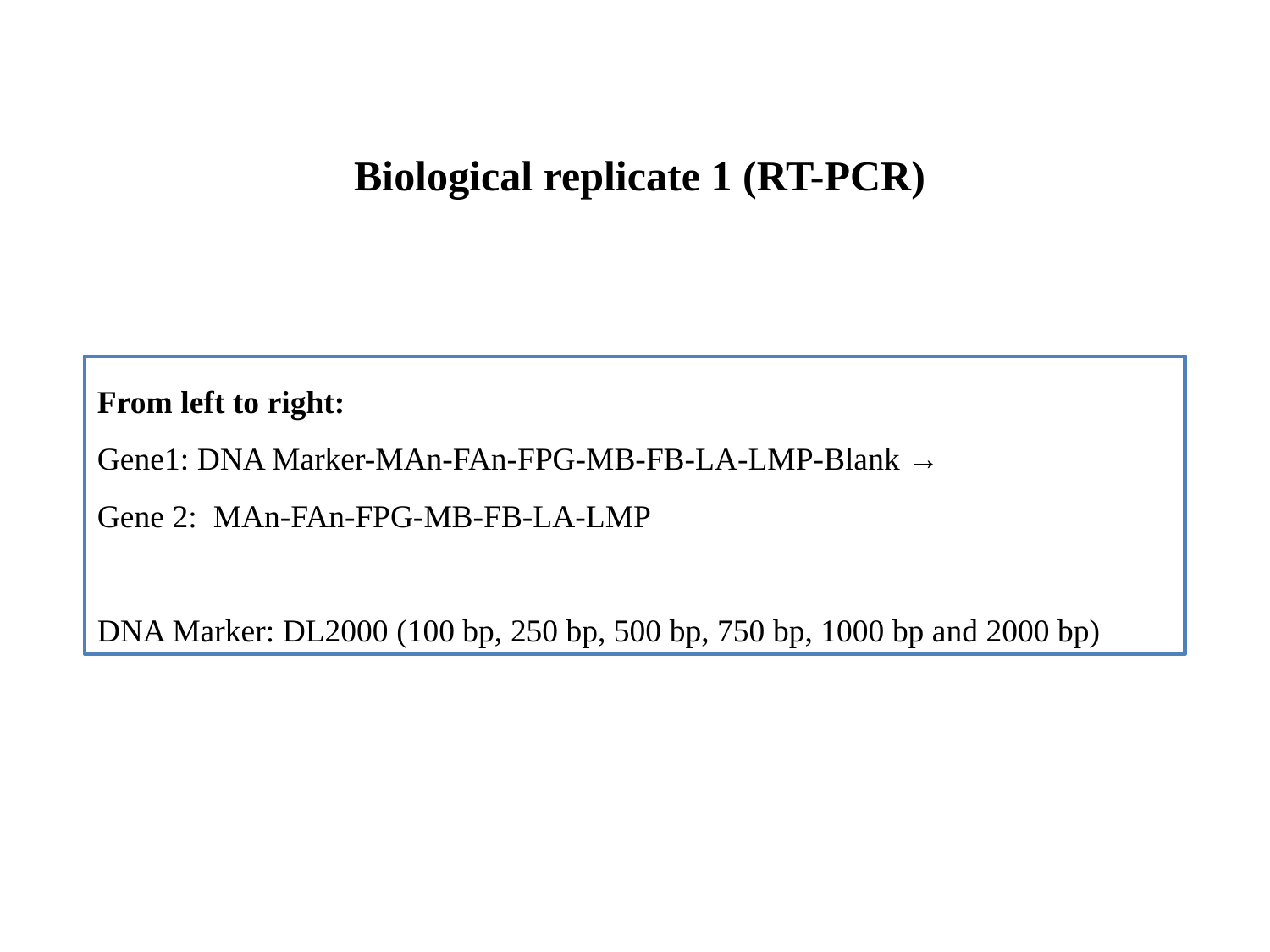

Biological replicate 1 (RT-PCR)
From left to right:
Gene1: DNA Marker-MAn-FAn-FPG-MB-FB-LA-LMP-Blank →
Gene 2: MAn-FAn-FPG-MB-FB-LA-LMP
DNA Marker: DL2000 (100 bp, 250 bp, 500 bp, 750 bp, 1000 bp and 2000 bp)

## Slide 2
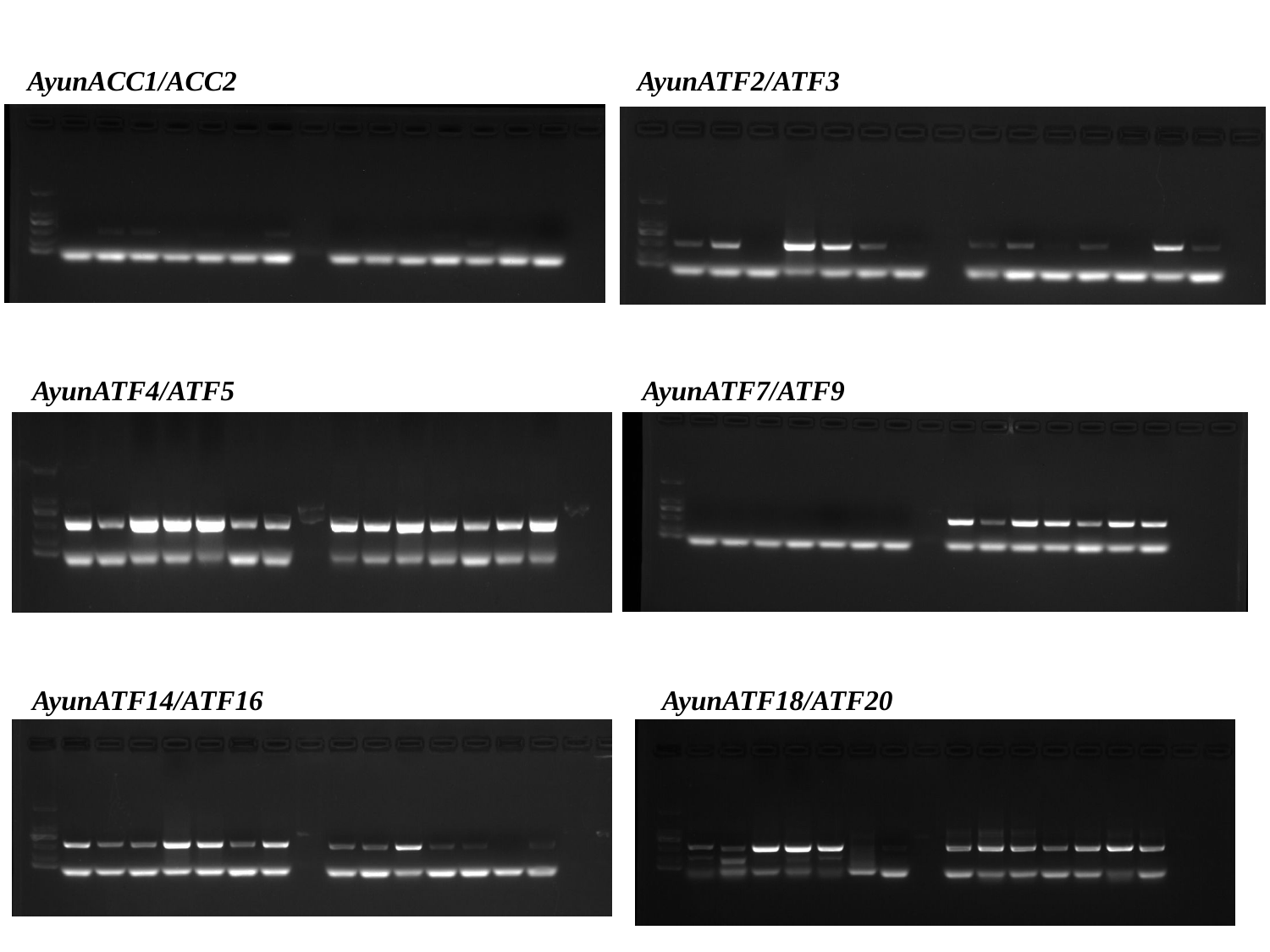

AyunACC1/ACC2
AyunATF2/ATF3
AyunATF4/ATF5
AyunATF7/ATF9
AyunATF18/ATF20
AyunATF14/ATF16

## Slide 3
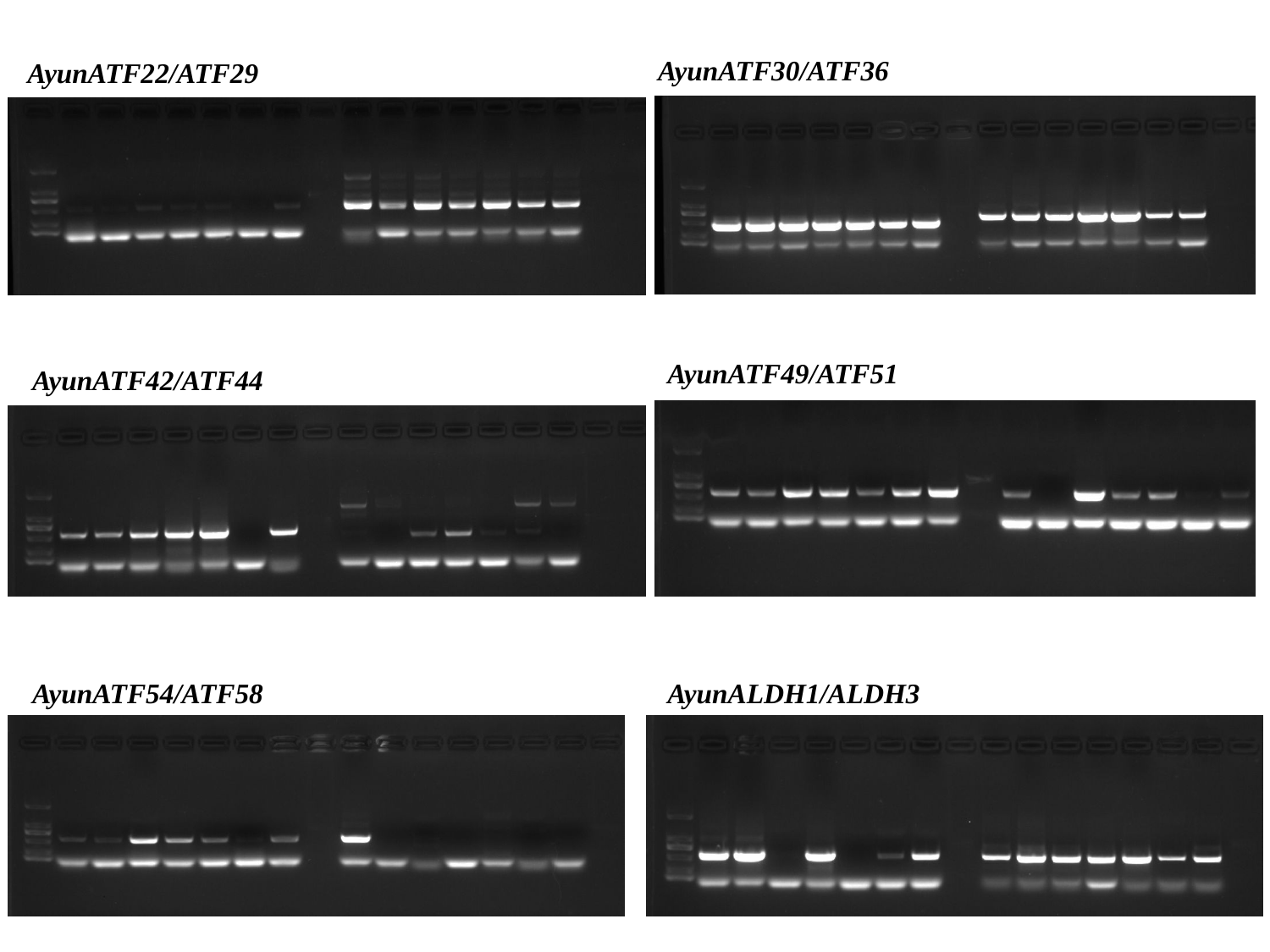

AyunATF30/ATF36
AyunATF22/ATF29
AyunATF49/ATF51
AyunATF42/ATF44
AyunATF54/ATF58
AyunALDH1/ALDH3

## Slide 4
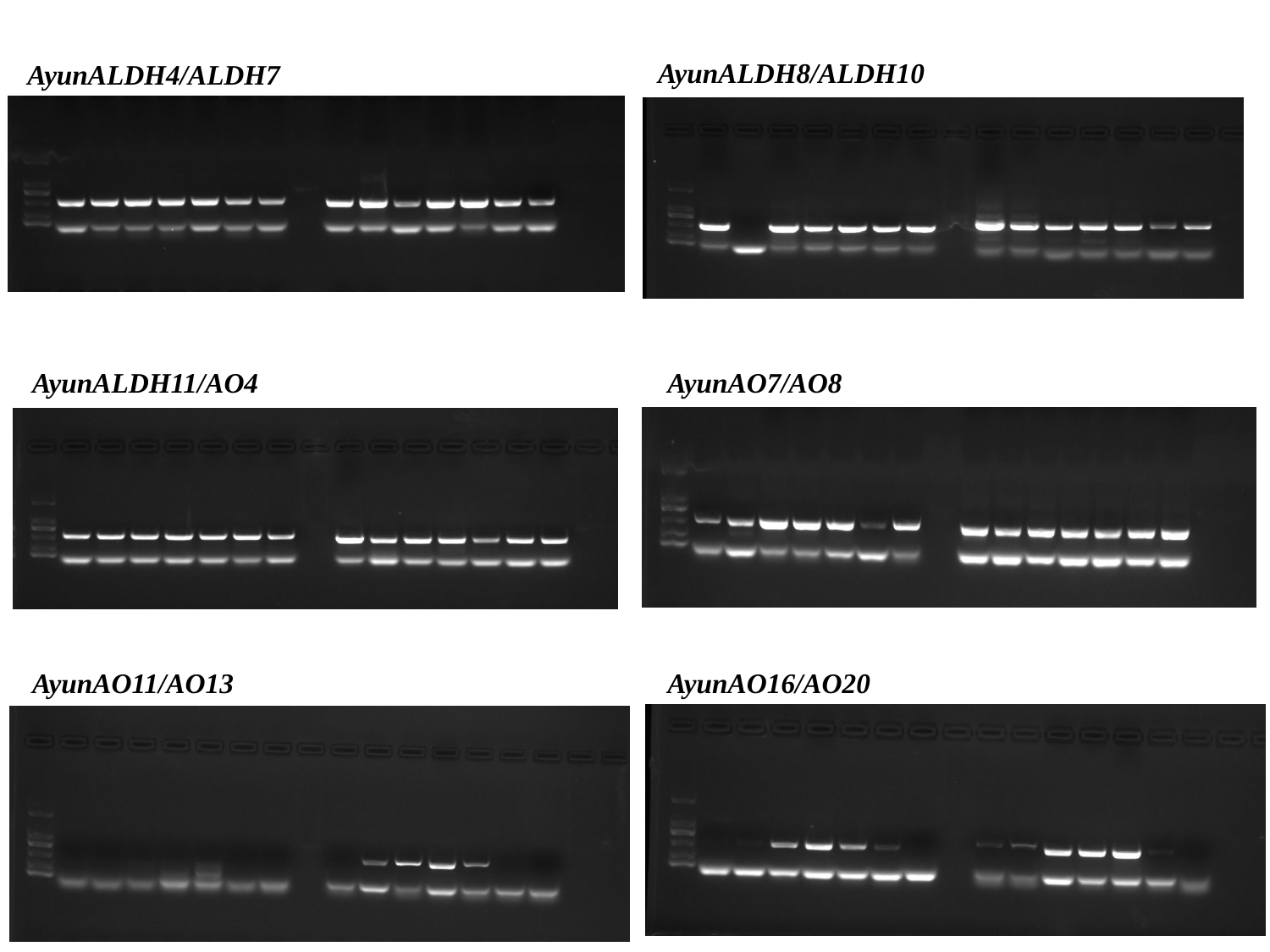

AyunALDH8/ALDH10
AyunALDH4/ALDH7
AyunALDH11/AO4
AyunAO7/AO8
AyunAO11/AO13
AyunAO16/AO20

## Slide 5
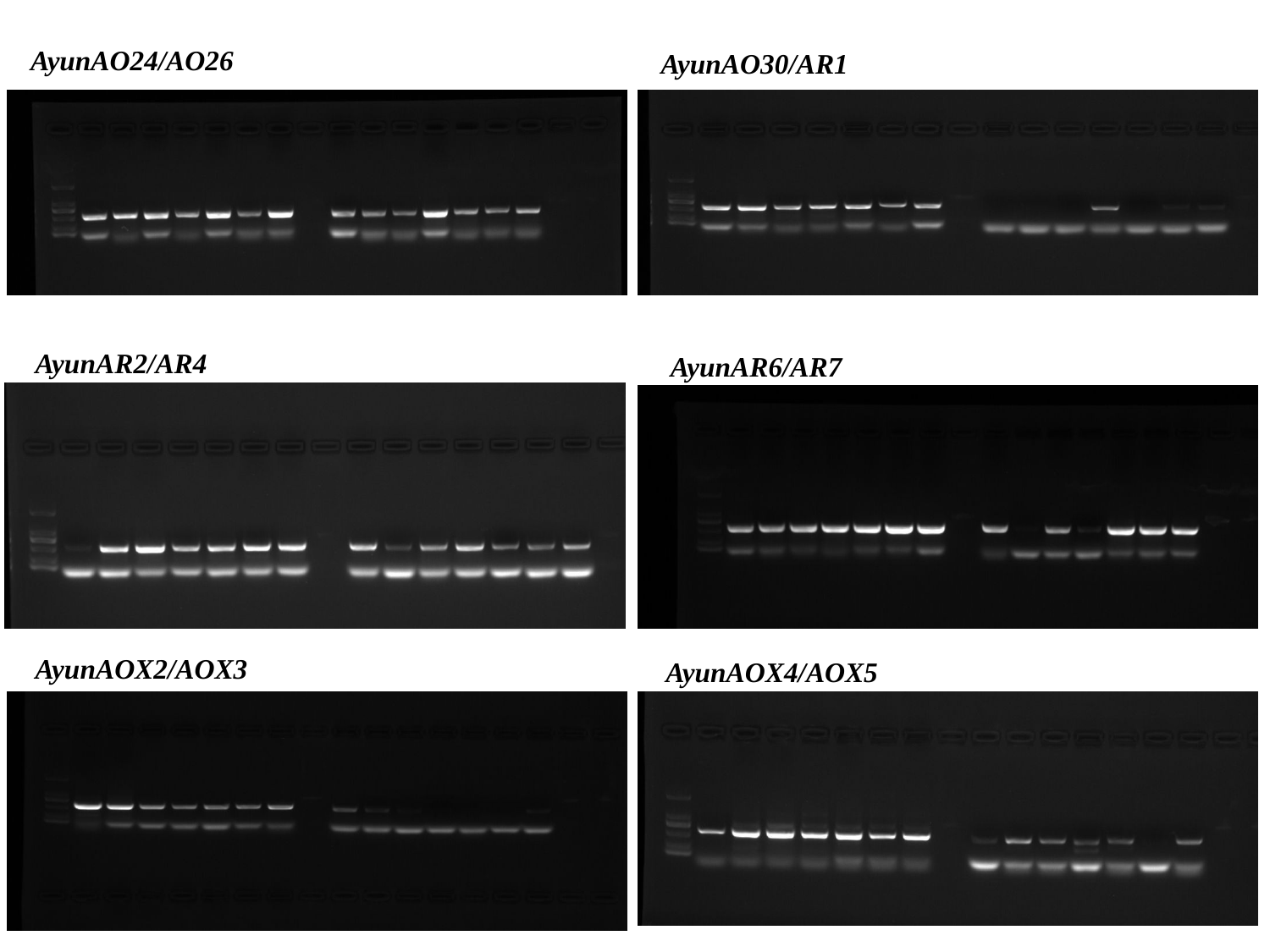

AyunAO24/AO26
AyunAO30/AR1
AyunAR2/AR4
AyunAR6/AR7
AyunAOX2/AOX3
AyunAOX4/AOX5

## Slide 6
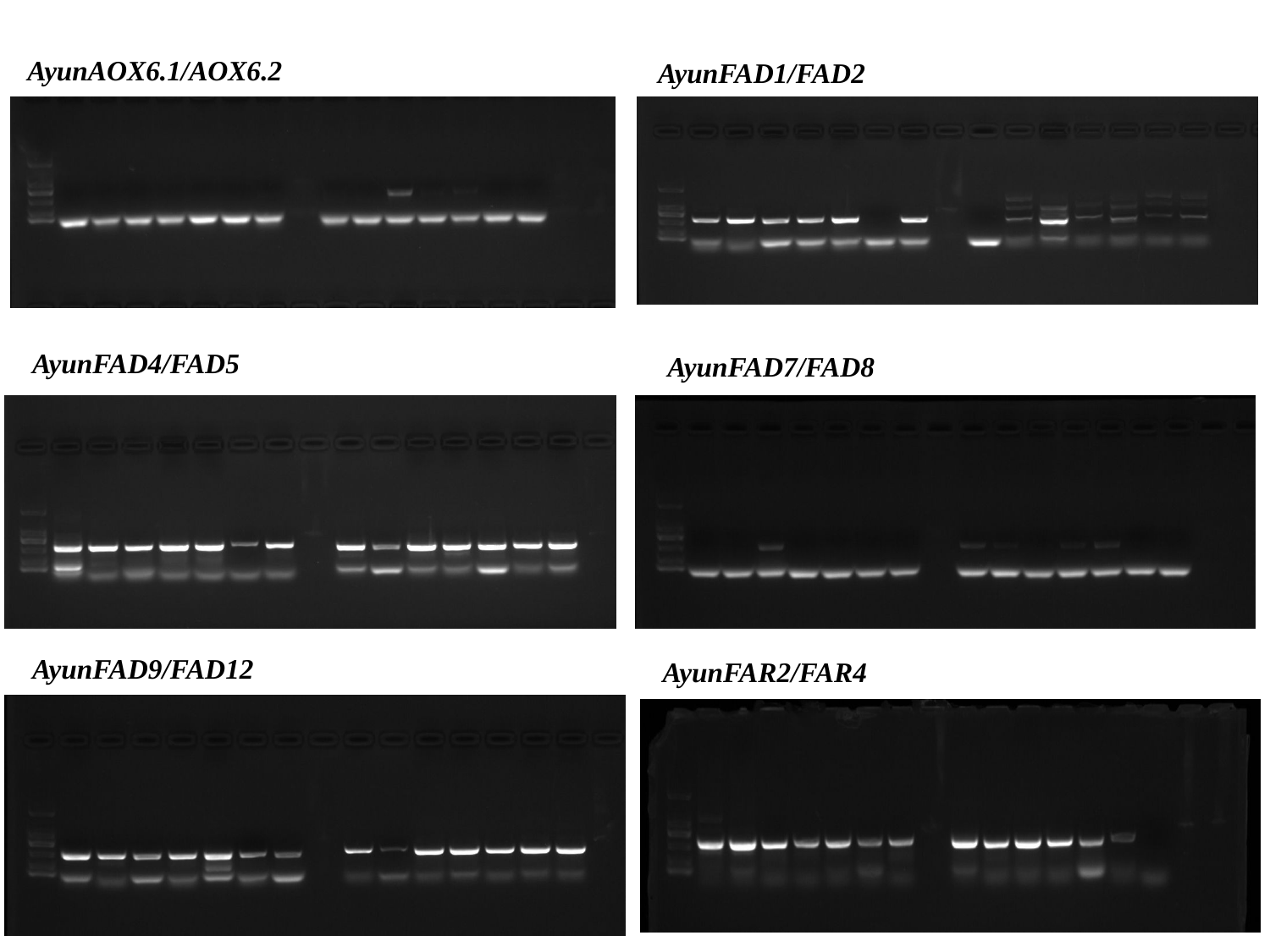

AyunAOX6.1/AOX6.2
AyunFAD1/FAD2
AyunFAD4/FAD5
AyunFAD7/FAD8
AyunFAD9/FAD12
AyunFAR2/FAR4

## Slide 7
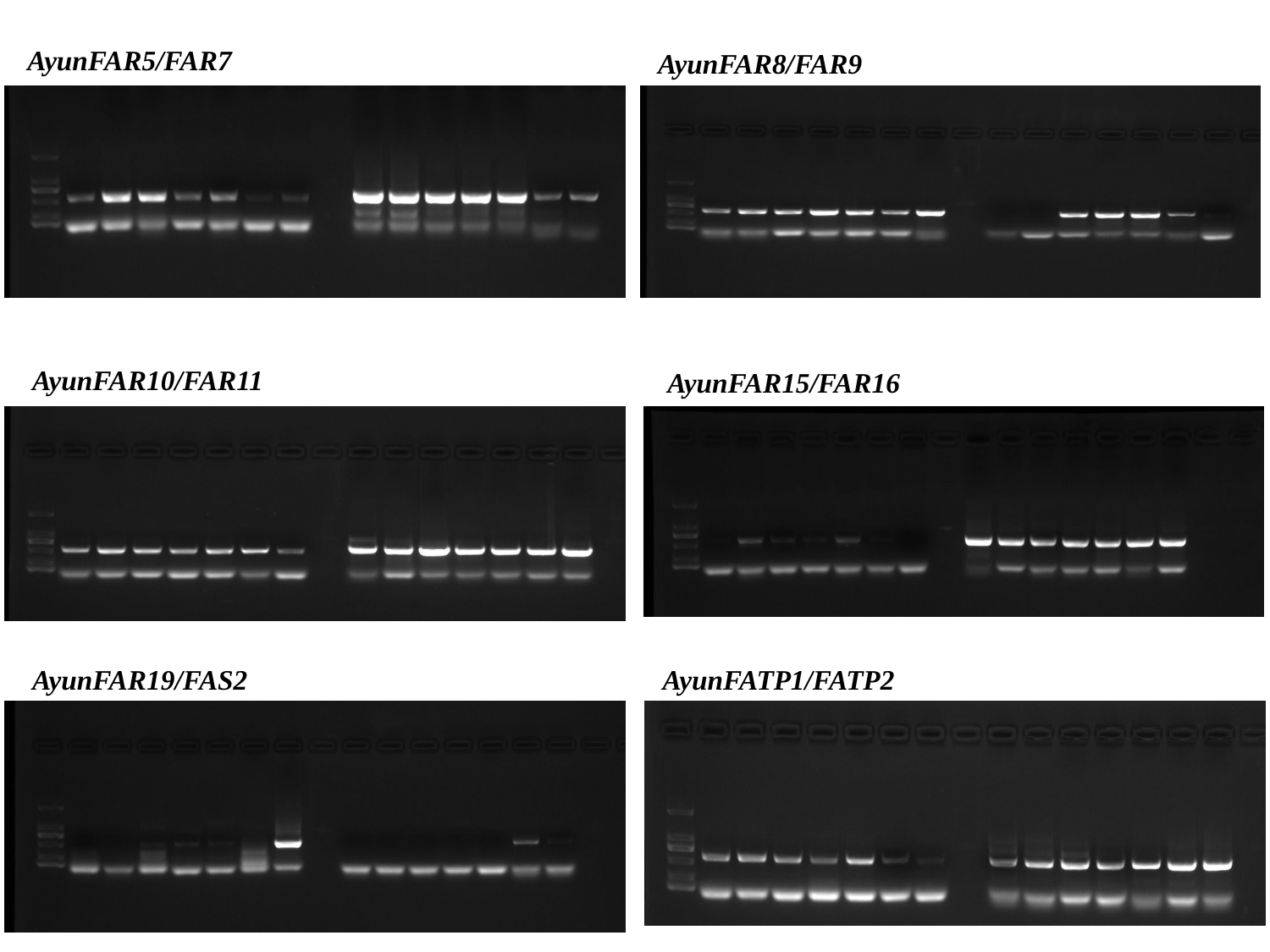

AyunFAR5/FAR7
AyunFAR8/FAR9
AyunFAR10/FAR11
AyunFAR15/FAR16
AyunFAR19/FAS2
AyunFATP1/FATP2

## Slide 8
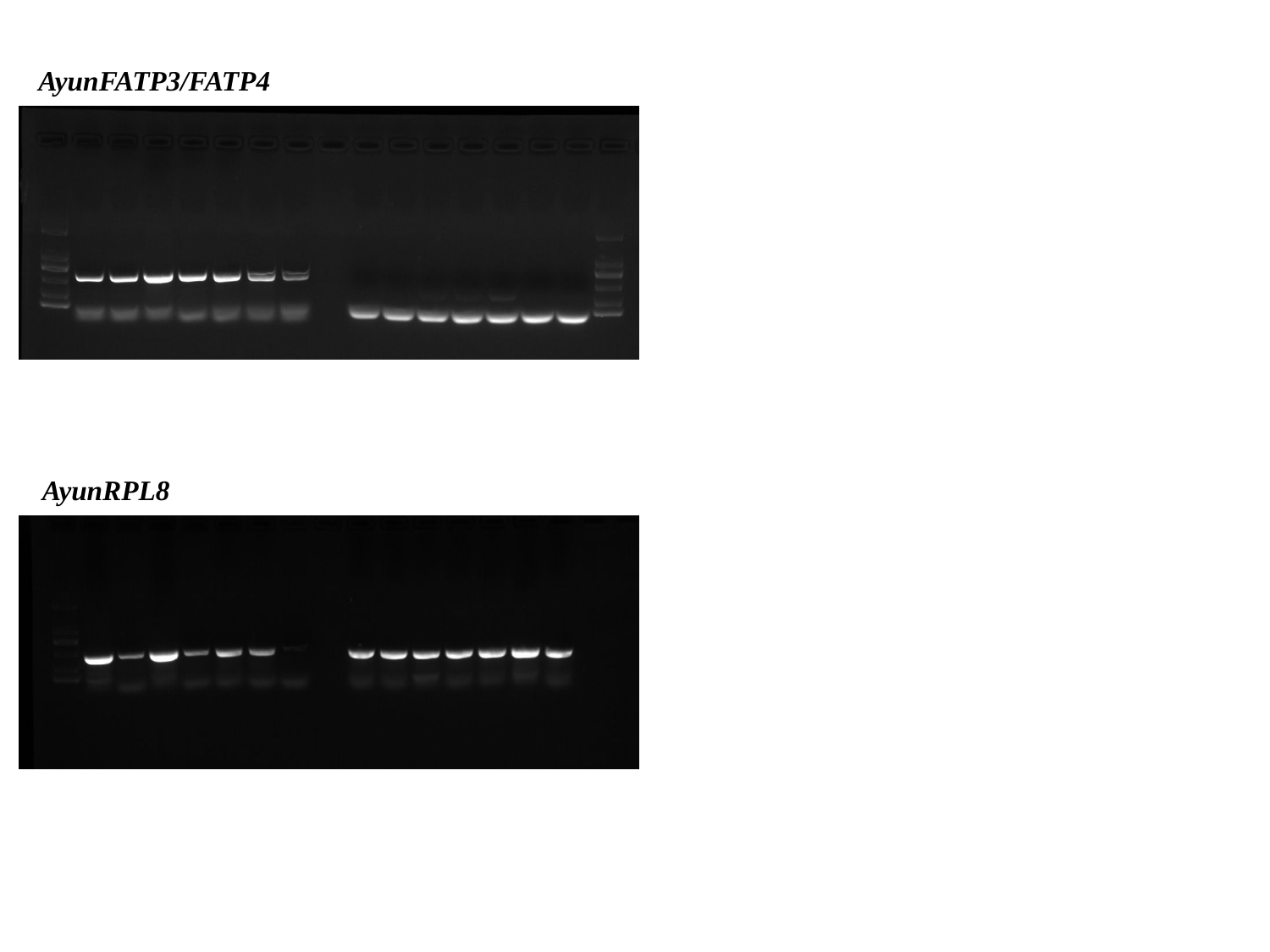

AyunFATP3/FATP4
AyunRPL8

## Slide 9
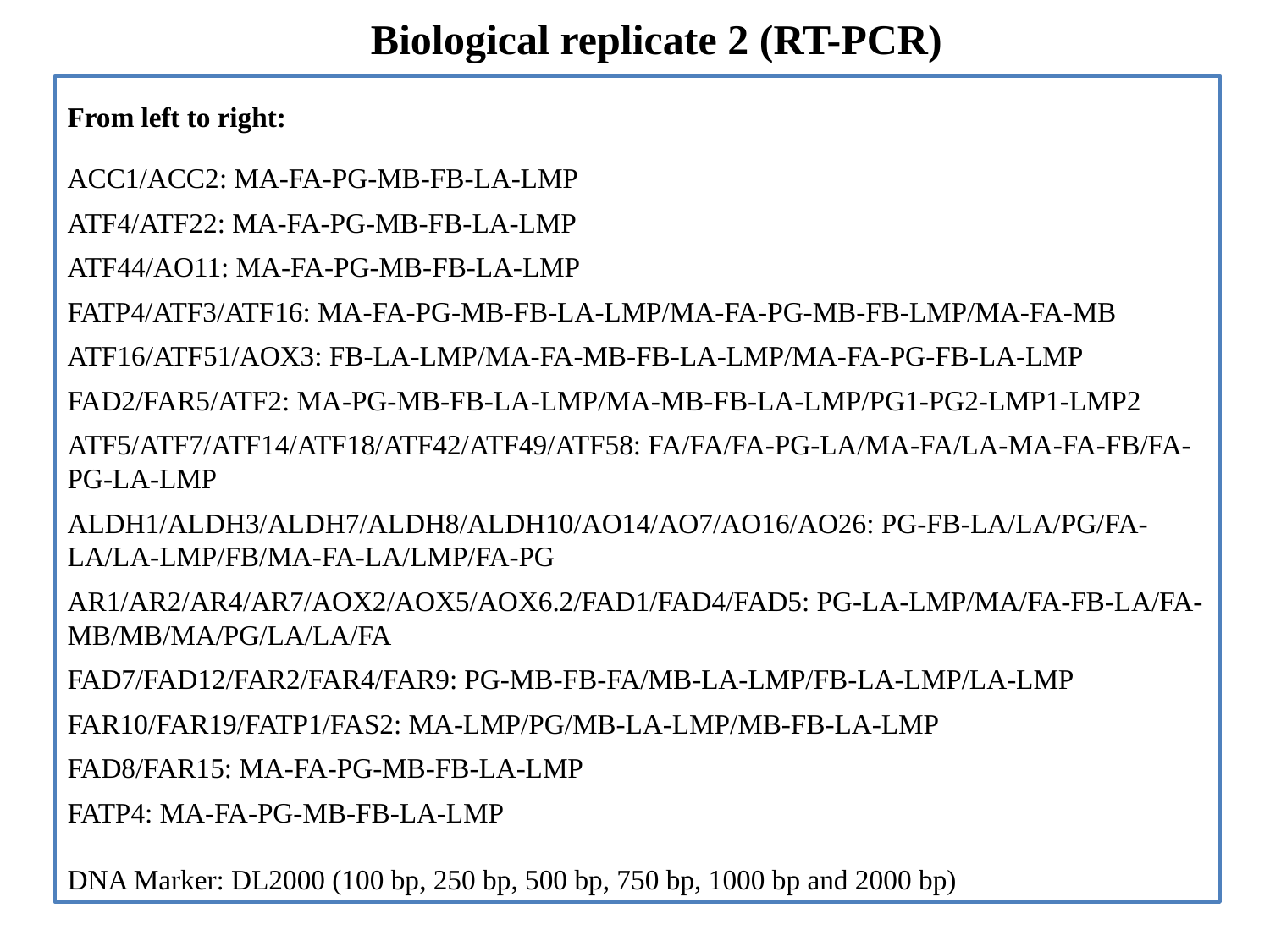

Biological replicate 2 (RT-PCR)
From left to right:
ACC1/ACC2: MA-FA-PG-MB-FB-LA-LMP
ATF4/ATF22: MA-FA-PG-MB-FB-LA-LMP
ATF44/AO11: MA-FA-PG-MB-FB-LA-LMP
FATP4/ATF3/ATF16: MA-FA-PG-MB-FB-LA-LMP/MA-FA-PG-MB-FB-LMP/MA-FA-MB
ATF16/ATF51/AOX3: FB-LA-LMP/MA-FA-MB-FB-LA-LMP/MA-FA-PG-FB-LA-LMP
FAD2/FAR5/ATF2: MA-PG-MB-FB-LA-LMP/MA-MB-FB-LA-LMP/PG1-PG2-LMP1-LMP2
ATF5/ATF7/ATF14/ATF18/ATF42/ATF49/ATF58: FA/FA/FA-PG-LA/MA-FA/LA-MA-FA-FB/FA-PG-LA-LMP
ALDH1/ALDH3/ALDH7/ALDH8/ALDH10/AO14/AO7/AO16/AO26: PG-FB-LA/LA/PG/FA-LA/LA-LMP/FB/MA-FA-LA/LMP/FA-PG
AR1/AR2/AR4/AR7/AOX2/AOX5/AOX6.2/FAD1/FAD4/FAD5: PG-LA-LMP/MA/FA-FB-LA/FA-MB/MB/MA/PG/LA/LA/FA
FAD7/FAD12/FAR2/FAR4/FAR9: PG-MB-FB-FA/MB-LA-LMP/FB-LA-LMP/LA-LMP
FAR10/FAR19/FATP1/FAS2: MA-LMP/PG/MB-LA-LMP/MB-FB-LA-LMP
FAD8/FAR15: MA-FA-PG-MB-FB-LA-LMP
FATP4: MA-FA-PG-MB-FB-LA-LMP
DNA Marker: DL2000 (100 bp, 250 bp, 500 bp, 750 bp, 1000 bp and 2000 bp)

## Slide 10
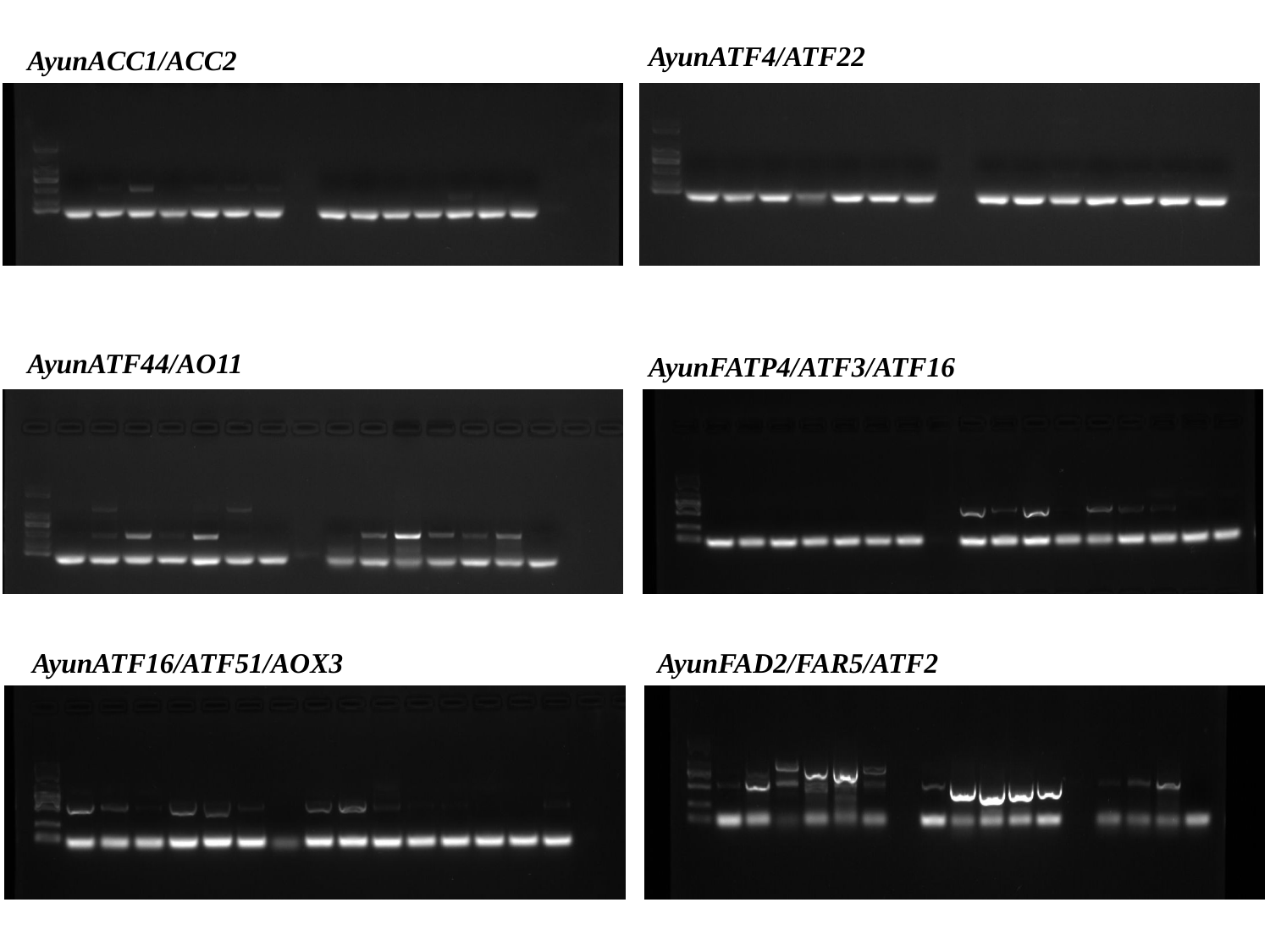

AyunATF4/ATF22
AyunACC1/ACC2
AyunATF44/AO11
AyunFATP4/ATF3/ATF16
AyunATF16/ATF51/AOX3
AyunFAD2/FAR5/ATF2

## Slide 11
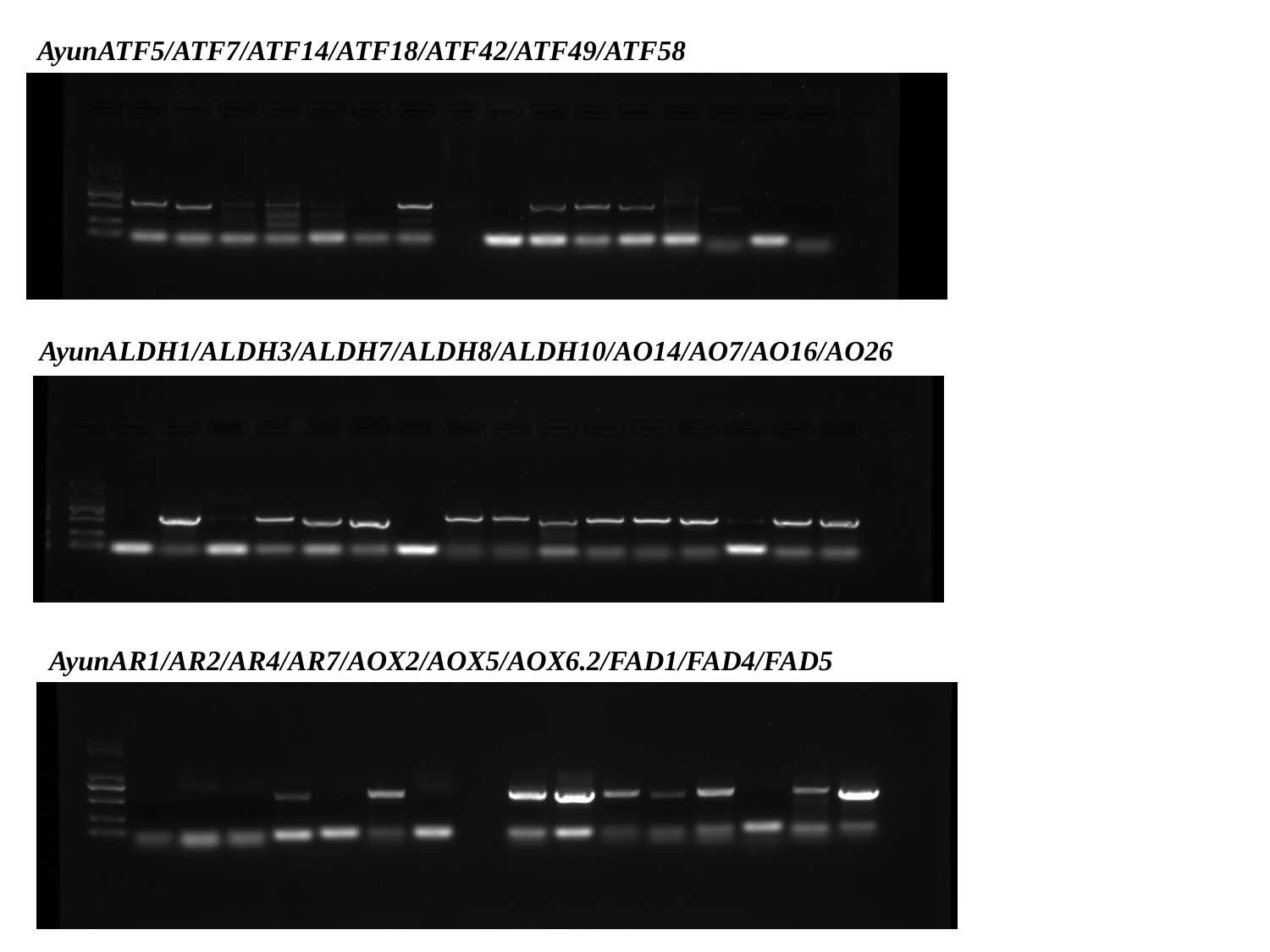

AyunATF5/ATF7/ATF14/ATF18/ATF42/ATF49/ATF58
AyunALDH1/ALDH3/ALDH7/ALDH8/ALDH10/AO14/AO7/AO16/AO26
AyunAR1/AR2/AR4/AR7/AOX2/AOX5/AOX6.2/FAD1/FAD4/FAD5

## Slide 12
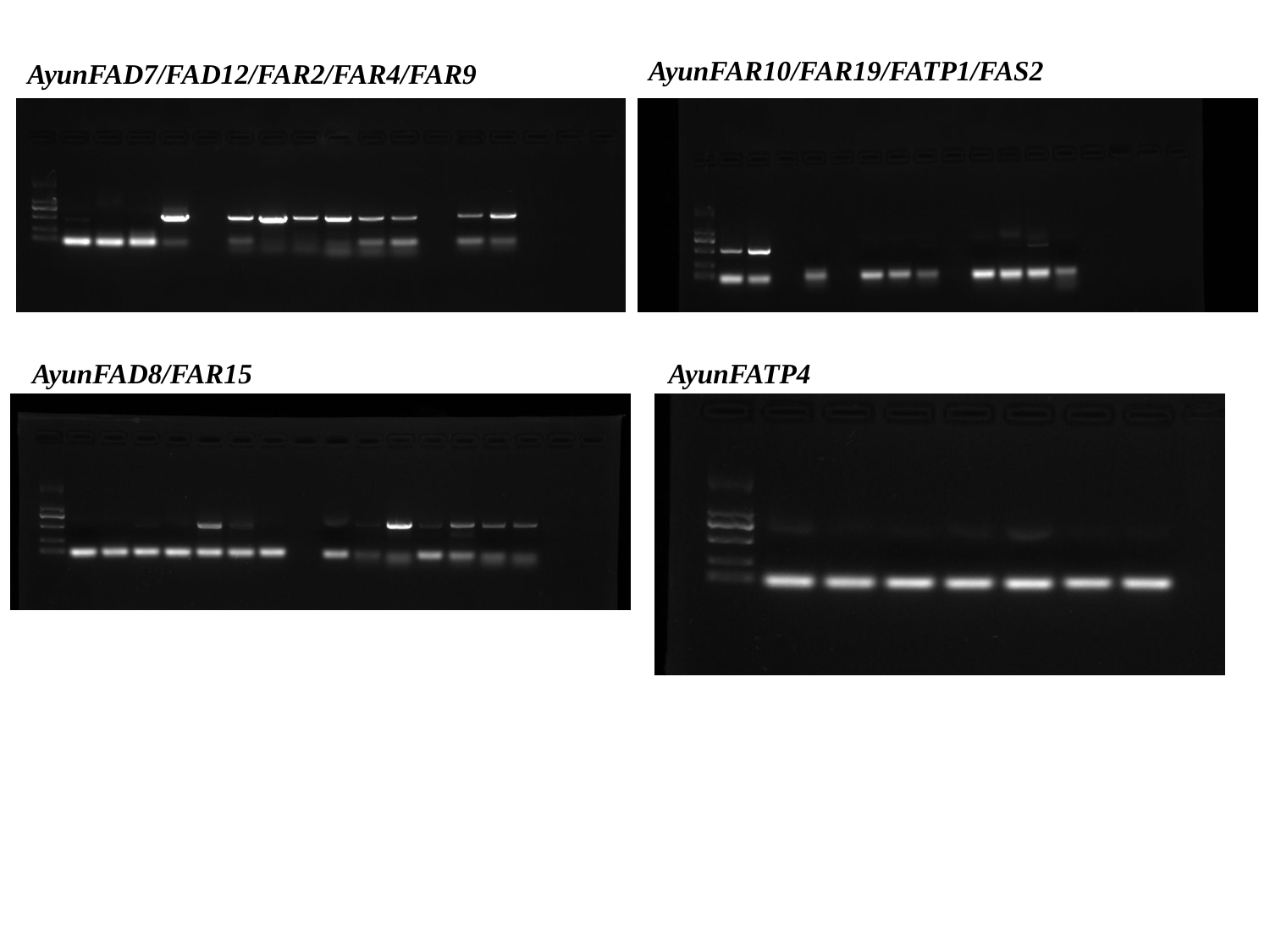

AyunFAR10/FAR19/FATP1/FAS2
AyunFAD7/FAD12/FAR2/FAR4/FAR9
AyunFAD8/FAR15
AyunFATP4

## Slide 13
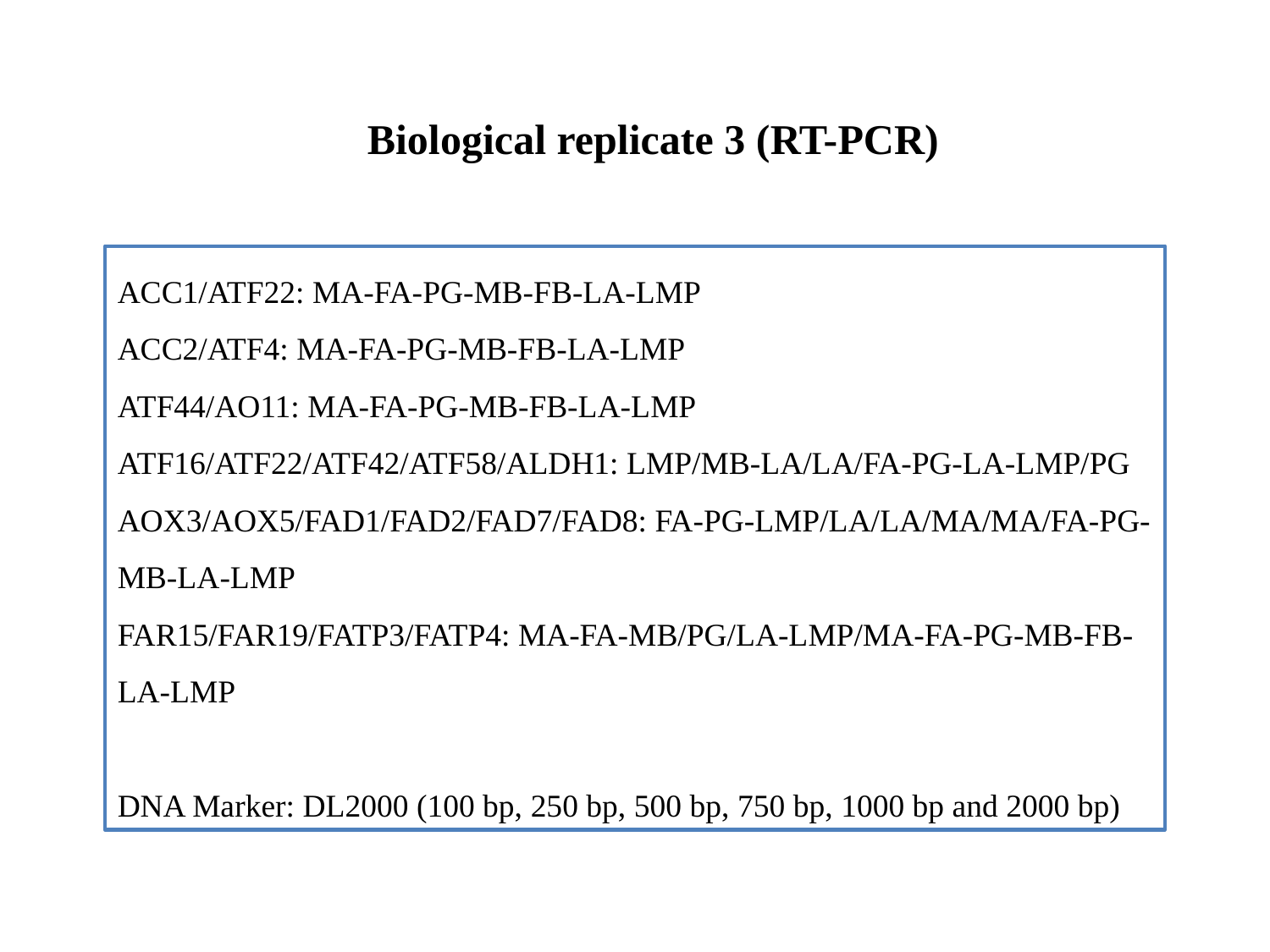

Biological replicate 3 (RT-PCR)
ACC1/ATF22: MA-FA-PG-MB-FB-LA-LMP
ACC2/ATF4: MA-FA-PG-MB-FB-LA-LMP
ATF44/AO11: MA-FA-PG-MB-FB-LA-LMP
ATF16/ATF22/ATF42/ATF58/ALDH1: LMP/MB-LA/LA/FA-PG-LA-LMP/PG
AOX3/AOX5/FAD1/FAD2/FAD7/FAD8: FA-PG-LMP/LA/LA/MA/MA/FA-PG-MB-LA-LMP
FAR15/FAR19/FATP3/FATP4: MA-FA-MB/PG/LA-LMP/MA-FA-PG-MB-FB-LA-LMP
DNA Marker: DL2000 (100 bp, 250 bp, 500 bp, 750 bp, 1000 bp and 2000 bp)

## Slide 14
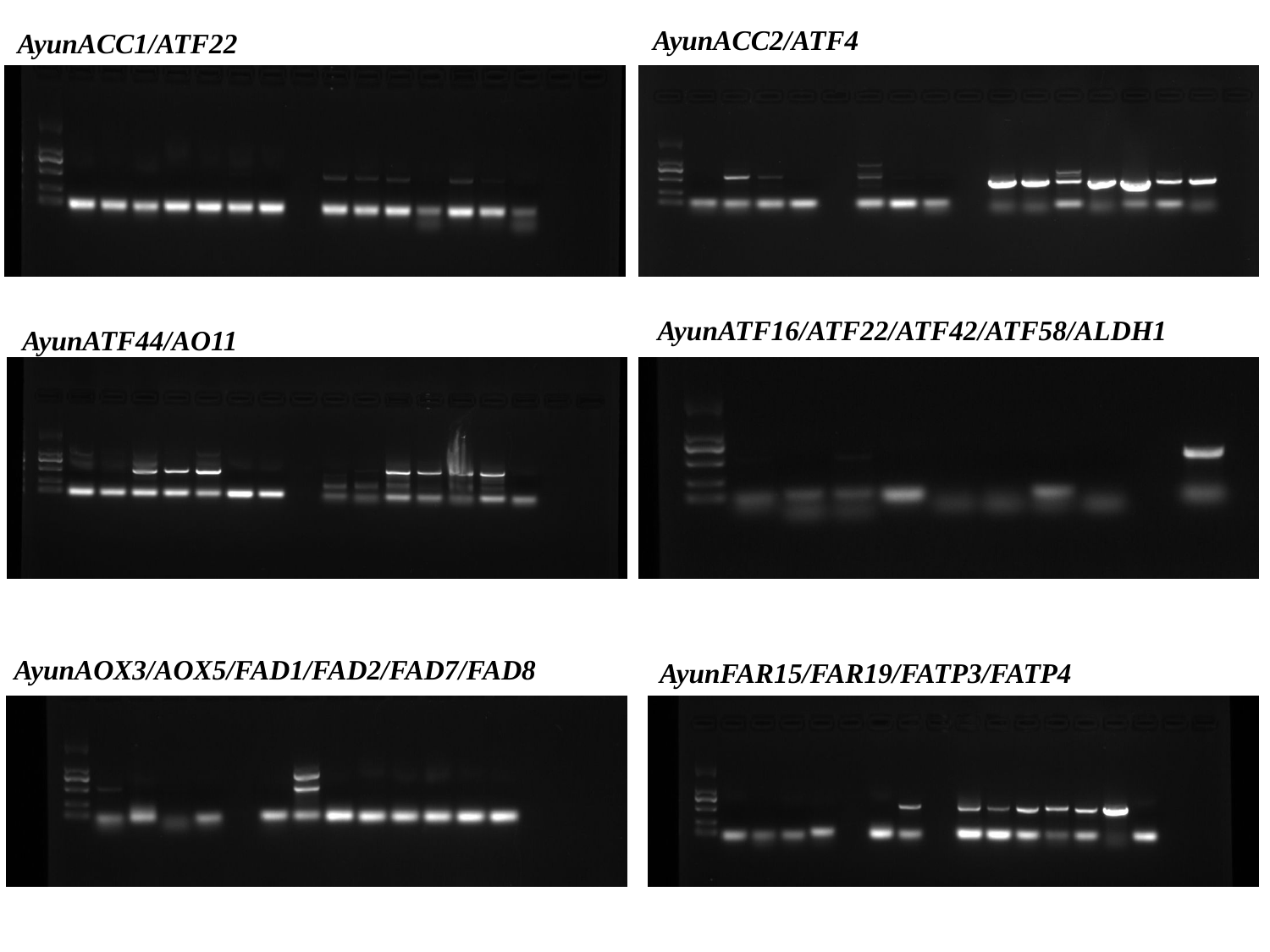

AyunACC2/ATF4
AyunACC1/ATF22
AyunATF16/ATF22/ATF42/ATF58/ALDH1
AyunATF44/AO11
AyunAOX3/AOX5/FAD1/FAD2/FAD7/FAD8
AyunFAR15/FAR19/FATP3/FATP4

## Slide 15
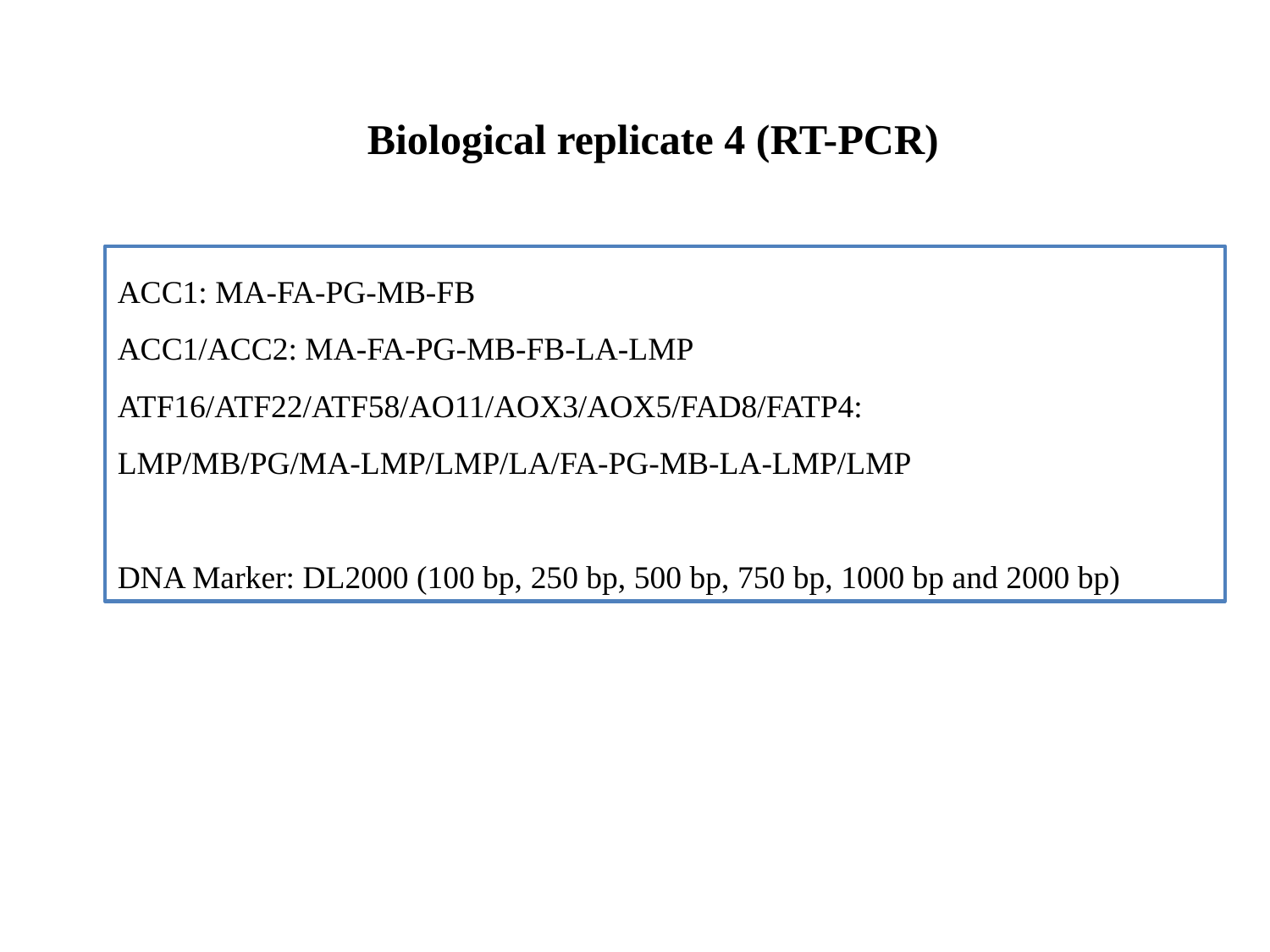

Biological replicate 4 (RT-PCR)
ACC1: MA-FA-PG-MB-FB
ACC1/ACC2: MA-FA-PG-MB-FB-LA-LMP
ATF16/ATF22/ATF58/AO11/AOX3/AOX5/FAD8/FATP4: LMP/MB/PG/MA-LMP/LMP/LA/FA-PG-MB-LA-LMP/LMP
DNA Marker: DL2000 (100 bp, 250 bp, 500 bp, 750 bp, 1000 bp and 2000 bp)

## Slide 16
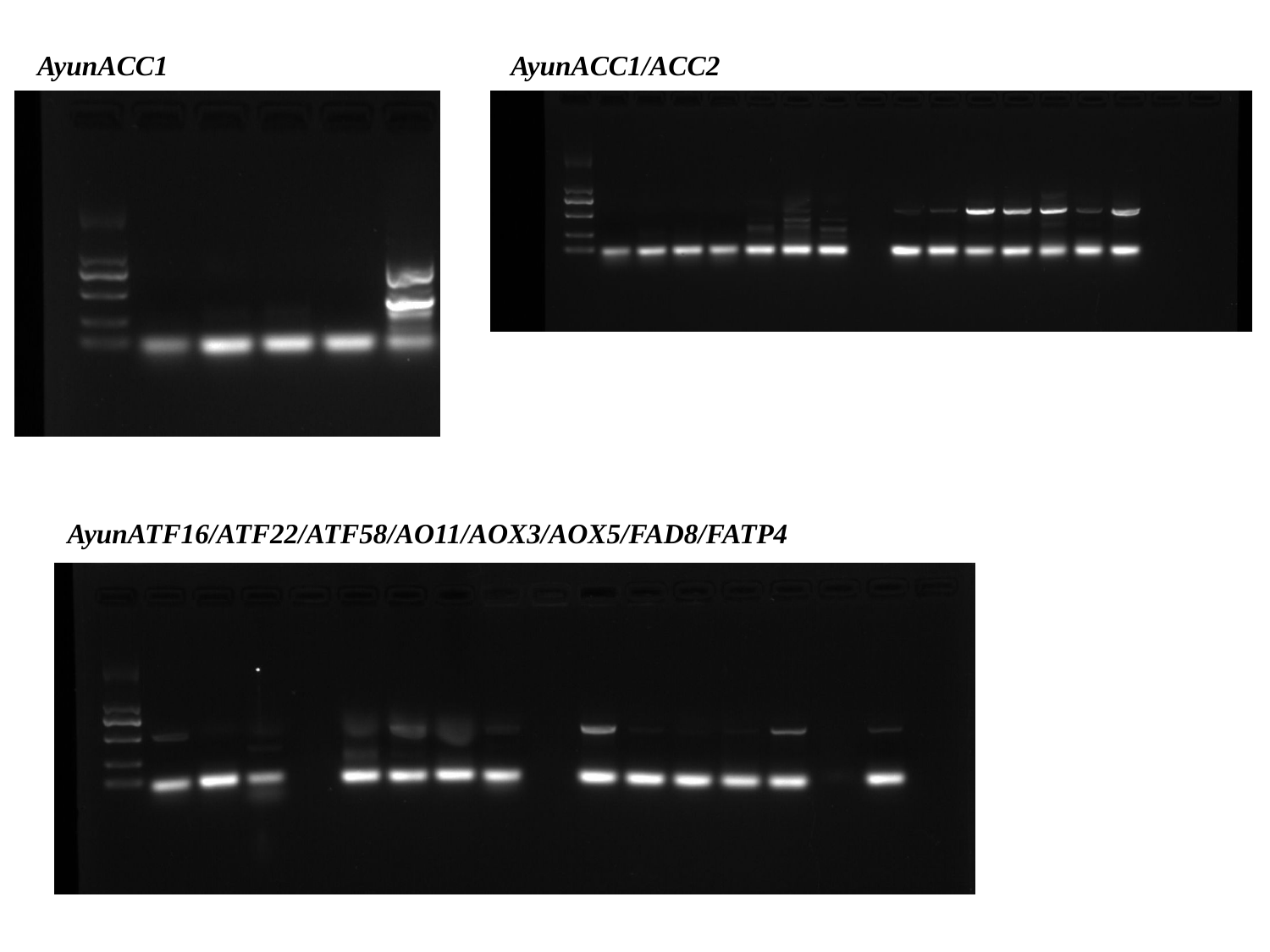

AyunACC1
AyunACC1/ACC2
AyunATF16/ATF22/ATF58/AO11/AOX3/AOX5/FAD8/FATP4
